# What Is Central to Political Belief System Networks?

**DOI:** 10.1177/0146167218824354

**Published:** 2019-01-28

**Authors:** Mark J. Brandt, Chris G. Sibley, Danny Osborne

**Affiliations:** 1 Tilburg University, The Netherlands; 2 The University of Auckland, New Zealand

**Keywords:** belief systems, identity, networks, political policies, political psychology

## Abstract

A central challenge for identifying core components of a belief system is examining the position of components within the structure of the entire belief system. We test whether operational (i.e., positions on issues) or symbolic (i.e., affective attachments to political groups and labels) components are most central by modeling a political belief system as a network of interconnected attitudes and beliefs. Across seven waves of representative panel data from New Zealand, we find that symbolic components are more central than operational components (*ds* range = 0.78-0.97). Symbolic components were also closer than operational components in the network to self-reported voting (*d* = −2.43), proenvironmental actions (*ds* = −1.71 and −1.63), and religious behaviors (*d* = −0.74). These findings are consistent with perspectives that emphasize the importance of symbolic politics in tying belief systems together and motivating behavior, and further the link between political belief system research and network science.

Political belief systems lay at the heart of multiple disciplines including social psychology, political science, and sociology. They can encourage or obstruct social movements and social changes as they reflect people’s social circumstances and provide people with a lens to view the world (Duckitt & Sibley, 2010; Jost, Nosek, & Gosling, 2008; Kahan, Peters, Dawson, & Slovic, 2017). Although different disciplines, and even scholars within the same discipline, have different definitions for political belief systems and related terms (e.g., ideology, worldview), they tend to converge on the idea that belief systems are the interrelationships of attitudes and beliefs relevant to politics (Gerring, 1997). In this article, we integrate research that models psychological phenomena as networks with the idea that belief systems are defined by the interrelationships of attitudes and beliefs to understand what is central to political belief systems. Identifying the central components of political belief systems is important because it informs us about how people reason about political issues (Bartels, 2002; Hatemi & McDermott, 2016; Kahan et al., 2017) and the components most likely to affect their political decisions (Campbell, Converse, Miller, & Stokes, 1960; Converse, 1964, 2000; Ellis & Stimson, 2012; Kinder & Kalmoe, 2017). A deeper understanding of the central feature (or features) of political belief systems would also inform people’s reactions to political events (Huddy, Mason, & Aarøe, 2015), and their positions on new policy proposals (Cohen, 2003; Malka & Lelkes, 2010).

## Operational or Symbolic Components as Central?

Given these implications, scholars from multiple disciplines over the last six decades have worked to identify if the operational components (i.e., positions on specific issues, such as government spending) or symbolic components (i.e., affective attachments to political groups and labels, such as ideological identification or party identification) are most central to political belief systems. Said another way, are political issues or political identities at the center of political belief systems? Traditionally, the centrality question is tested by examining how strongly operational and symbolic measures are associated with key outcomes, such as voting behavior (Campbell et al., 1960; Jost, 2006; Kinder & Kalmoe, 2017) and negative views of ideological rivals (Mason, 2018); how operational and symbolic measures respond to experimental manipulations of the political context (Cohen, 2003; Huddy et al., 2015; Malka & Lelkes, 2010); or by testing if symbolic measures are more likely to cause changes to (and therefore be more central to) operational measures and vice versa (Chen & Goren, 2016; Converse, 1964; Highton & Kam, 2011; Jackson, 1975). A central challenge for these methods of identifying core components of a belief system is examining the position of the components within the structure of the belief system as a whole. Because prior methods do not overcome this challenge, the relationships identified in past research may be on the periphery of the system.

## Belief Systems as Networks

One solution to this challenge is to directly model the interrelationships of attitudes and beliefs relevant to politics (i.e., the political belief system) as a network of interacting nodes (see Boutyline & Vaisey, 2017 for the first use of this general approach). We treat each node as a measure of an operational or symbolic component of the belief system. The belief system then consists of all of the relevant nodes and their connections with one another. This approach integrates work distinguishing symbolic and operational components of belief systems (e.g., Ellis & Stimson, 2012; Sears, 1993) with research modeling psychopathologies (Borsboom & Cramer, 2013), personality (Cramer et al., 2012), and single attitudes (Dalege et al., 2015) as networks. It has several distinct advantages.

First, a network approach successfully operationalizes the definition of political belief systems. By modeling belief systems as a network, we can explicitly model the interrelationships between the attitudes and beliefs relevant to politics.

Second, the network approach easily accommodates many belief system components within the analysis. Specifically, multiple operational and symbolic beliefs, as well as their interrelationships, can be modeled simultaneously. This allows for the possible recognition of multiple central and peripheral constructs, as well as constructs that might be somewhere in the middle of the central–peripheral continuum.

Third, by modeling belief systems as networks, we can adopt measures of network centrality from network science that are used to assess centrality within the structure of a broad array of systems (Barrat, Barthelemy, Pastor-Satorras, & Vespignani, 2004; Borgatti, 2005; Newman, 2010; Opsahl, Agneessens, & Skvoretz, 2010). Because belief systems are interrelationships between attitudes and beliefs, measures of centrality should take into account where a component is within that web of interrelationships. Centrality indicators from network science do just that, by identifying how a particular node is embedded within the network.

We focus on three centrality indicators that have been used in past work on psychological networks (e.g., Boutyline & Vaisey, 2017; Costantini, Epskamp, et al., 2015; cf. Freeman, 1978): strength, closeness, and betweenness centrality. *Strength centrality* is the sum of the absolute value of the edge weights that directly connect to a node (Barrat et al., 2004; Newman, 2010), and is an indicator of the immediate connections and potential influences a belief system component has on its neighbors. The other two measures of centrality are related to the position of the node within the overall structure of the belief system network. *Closeness centrality* is the inverse of the sum of the distance between a node and all other nodes (Borgatti, 2005), thereby representing how “quickly” the influence of a particular component can get from one component of the belief system to the rest of the components in the system. *Betweenness centrality* is the number of shortest paths that pass through a node between two other nodes (Borgatti, 2005). Higher betweenness thus captures how necessary the belief system component is for linking together the other parts of the belief system.

Although strength centrality may be an important property of belief system networks, closeness and betweenness centrality are theoretically most closely related to belief system scholars’ understanding of centrality. Whereas strength centrality taps into the embeddedness of a belief system component in its particular region of the belief system, closeness and betweenness centrality assess how well a component can tie components from disparate regions of the belief system together and influence the network as a whole. This is consistent with Converse’s (1964) description of the centrality of belief system components as “the role that they play in the belief system *as a whole*” (p. 208, emphasis added). For example, a component with high betweenness can serve as a bridge between different regions of the belief system, thereby helping to form a single belief system rather than multiple nonoverlapping belief systems. This is different from other descriptions of centrality, including those provided by Converse, that identify central variables as being more causally potent. Causal potency does not necessarily say anything about centrality, as the causally potent variable may be on the periphery of the belief system. Only by modeling the whole system as a network are we able to locate components within the broader belief system.

## Hypotheses

Critically, modeling belief systems as networks enables us to test two key hypotheses. The *operational centric* hypothesis predicts that operational components of the belief system (i.e., support or opposition for specific policy issues) will be the most central. This is supported by work showing that people adopt party affiliations (Chen & Goren, 2016) and ideological identifications (e.g., identification as right wing or left wing; De Vries, Hakhverdian, & Lancee, 2013) to fit their issue positions and that issue positions are reliably associated with vote choice (Ansolabehere, Rodden, & Snyder, 2008), especially for competitive elections (Lachat, 2011). Conversely, the *symbolic centric* hypothesis predicts that symbolic components of the belief system will be the most central. This is supported by work showing that people adopt issue positions that match their party affiliations (Cohen, 2003) or ideological identifications (Malka & Lelkes, 2010; see also Zaller, 1992) and that symbolic components of belief systems, such as party and ideological identification, are strongly associated with vote choice (Campbell et al., 1960; Green, Palmquist, & Schickler, 2002; Jost, 2006; Kinder & Kalmoe, 2017) and other political cognitions, emotions, and behaviors (Brandt, 2017; Huddy et al., 2015; Kahan et al., 2017; Mason, 2015, 2018). This hypothesis suggests that identities and symbolic attachments are at the core of belief systems and that support for specific issues emanate from them.

Although these two hypotheses are phrased in their starkest terms, it is important to recognize that centrality is not a binary designation within a network approach to belief systems. There are degrees of centrality. As such, even within symbolic and operational categories, there will be variation in the extent to which a particular node is central.

To help triangulate on the answer to our research question, we will also test how symbolic and operational components of the belief system are associated with political behaviors. Scholars have suggested that central (vs. peripheral) components of political belief systems are more strongly related to political behaviors (Campbell et al., 1960; Jost, 2006; Kinder & Kalmoe, 2017). The quintessential political behavior in democracies is voting. If symbolic or operational components tend to be more central than the other type of component, then the more central component should be closer in the network to voting behavior (Converse, 1964; Jost, 2006; Kinder & Kalmoe, 2017).

It may also be possible to extend this idea to other types of behavior that may not be so clearly relevant to the belief system. For example, although environmental behavior is not obviously tied to politics, environmental attitudes are often part of people’s political belief systems (Hornsey, Harris, Bain, & Fielding, 2016) and environmental behavior is often connected with people’s political views (Dietz, Stern, & Guagnano, 1998; Kahn, 2007; Kidwell, Farmer, & Hardesty, 2013; Scott & Willits, 1994; Van Liere & Dunlap, 1980). Similarly, although it is not necessary for religious behavior to be tied to political behavior, in practice, it appears that people’s religious and political belief systems are related (Brandt & Reyna, 2014; Malka & Soto, 2011; Mason & Wronski, 2018). Therefore, we also tested if the findings for voting behavior translate to other potentially relevant behavior, such as environmental and religious behavior. If the findings do extend to these domains, it provides suggestive evidence that what is central to belief systems is also central to nonpolitical, yet nonetheless consequential, behaviors.

## Prior Work on Political Belief System Networks

Although distinct from the aims of the current study, prior work has tested the related hypothesis about whether the American political belief system *developed* from ideological identification or authoritarian parenting values using a network approach (Boutyline & Vaisey, 2017). Using data from the 2000 American National Election Studies, these scholars find that ideological identification is the most betweenness-central component of the belief system, whereas authoritarian parenting values are on the periphery of the belief system. Given the assumptions specified in this prior work, this finding suggests that ideological identification is the developmental starting point of the American political belief system.

In this article, we extend this work in several ways. First, to test the operational centric and symbolic centric hypotheses, we focused our model of a political belief system network on both symbolic (party support, ideological identification) and operational (support for specific issues and policies) components. This allows us to focus on constructs core to the definition of political belief systems (Converse, 1964; Ellis & Stimson, 2012) that make up the bulk of the work teasing apart these hypotheses (e.g., Kinder & Kalmoe, 2017). Second, we extend our analysis of belief system centrality beyond indicators of network centrality to also investigate how closely symbolic and operational components are to voting behavior, a key political behavior, as well as extend this idea to self-reported environmental and religious behavior. Third, to model the complexity of belief systems in modern multiparty democracies, we analyzed data from the New Zealand attitudes and values study (NZAVS), a representative longitudinal panel study of the New Zealand voting age population between 2009 and 2016 (Sibley, 2014a, 2014b, 2014c) Fourth, and relatedly, we are able to examine how the models replicate across the years available in the NZAVS. Each wave contained between six and 10 items that assess symbolic components of political belief systems, and 16 and 30 items that assess operational components of political belief systems.

## Method

### Data

Waves 1 (2009) through 7 (2015) of the NZAVS were analyzed. The NZAVS was approved by The University of Auckland Human Participants Ethics Committee (Reference Numbers: 01488 and 6171). Participants with data on at least one belief system component in the wave were included in the analyses (see Table 1 for demographic information) and missing data were estimated using full information maximum likelihood estimation as a part of the analyses (see Table S1 in Supplemental Material for percentage of missing data per item). This survey is a multiyear longitudinal panel study based on a representative sample of New Zealand. For information about its representativeness and data collection procedures, see the survey’s extensive documentation (Sibley, 2014b, 2014c). For a brief summary of the political situation in New Zealand, see the Supplemental Materials.

**Table 1.** Demographic Information for the Seven Waves of the NZAVS Used in This Study.

Wave	*n*	*M* age	*SD* age	Women	Men
1	6,510	48.1	15.7	3,873	2,637
2	4,441	51.0	15.2	7,235	1,706
3	6,884	50.5	15.9	4,303	2,578
4	12,162	49.1	15.0	7,611	4,549
5	18,258	47.7	14.1	11,458	6,797
6	15,701	49.4	14.0	9,924	5,757
7	13,920	50.8	13.9	8,709	5,192

*Note*. Discrepancies between the number of women and men and the full sample are due to missing data. NZAVS = New Zealand attitudes and values study.

### Measures

For each wave of the survey, we identified all of the items assessing symbolic and operational components of the political belief system. Items were considered symbolic if they tapped into people’s support for, or identification with, political groups (i.e., parties) or labels (e.g., liberals; Ellis & Stimson, 2012). Items were considered operational if they tapped into people’s support for a policy that could be enacted politically (Ellis & Stimson, 2012). All items, their classification as symbolic or operational, and the wave(s) they were included in are found in Table 2. All items were coded so that higher scores represent more conservative positions in the New Zealand context. See Sibley (2014a) for the original materials for the NZAVS.

**Table 2.** The Items Analyzed and the Waves That Were Available.

Variables	Label	Wave 1	Wave 2	Wave 3	Wave 4	Wave 5	Wave 6	Wave 7	Total	Coding
Symbolic components										
Support for National Party	pNat	X	X	X	X	X	X	X	7	
Support for Labor Party	pLab	X	X	X	X	X	X	X	7	*R*
Support for Green Party	pGre	X	X	X	X	X	X	X	7	*R*
Support for ACT Party	pACT	X	X		X	X	X	X	6	
Support for Māori party	pMao	X	X	X	X	X	X	X	7	*R*
Support for United Future Party	pUni	X	X						2	
Support for New Zealand First Party	pNZ1				X	X	X	X	4	
Support for Mana Party	pMan					X	X		2	*R*
Support for Conservative Party	pCon					X	X		2	
Liberal/Conservative identification	IdID	X	X	X	X	X	X	X	7	
Right-wing/Left-wing identification	RwID			X	X	X	X	X	5	
Operational components										
Maori ownership of the seabed and foreshore	MSea	X	X	X	X	X	X	X	7	*R*
Reserving places for Maori students to study medicine	MMed	X	X	X	X	X	X	X	7	*R*
Rates exemptions on Maori land	MLnd	X	X	X	X	X	X	X	7	*R*
Crown (government) ownership of the seabed and foreshore	CSea	X	X	X	X	X	X	X	7	
Performance of the Haka at international sports events	Haka	X	X	X	X	X	X	X	7	*R*
Waitangi Day as a national celebration of biculturalism	WaiD	X	X	X	X	X	X	X	7	*R*
Teaching Maori language in New Zealand primary schools	Mlan	X	X	X	X	X	X	X	7	*R*
Singing the national anthem in Maori and English	NatA	X	X	X	X	X	X	X	7	*R*
Incentives to increase women’s participation in the paid workforce (paid for by government)	Winc	X	X	X	X	X	X	X	7	*R*
Introducing a program to enhance sustainable business growth among businesses owned and operated by women	Wbus	X	X	X	X	X	X	X	7	*R*
Affirmative action policies for women promoting entry into female underrepresented occupations, such as construction and the trades	WAA	X							1	*R*
The Civil Union Act	CiU	X	X	X	X	X	X		6	*R*
Same-sex marriage in New Zealand (The Marriage Amendment Act 2013)	SSM					X	X	X	3	*R*
The current antismacking bill. (i.e., it being illegal to smack children)	Asmk	X			X	X	X	X	5	*R*
Including religious instruction in Christianity as part of the school curriculum.	ReEd		X	X	X	X	X	X	6	
Legalized abortion for women, regardless of the reason	Aany			X	X	X	X	X	5	*R*
Legalized abortion when the woman’s life is endangered	Asp			X	X	X	X	X	5	*R*
A “flat” tax rate (everyone pays the same percentage of tax on their income)	Tax		X	X	X	X			4	
Policies promoting closer trade ties between India and New Zealand	IndT		X	X	X	X	X	X	6	*R*
Policies promoting closer trade ties between China and New Zealand	ChiT		X	X	X	X	X	X	6	*R*
Policies promoting more immigration from India to New Zealand	IndI		X	X	X	X	X	X	6	*R*
Policies promoting more immigration from China to New Zealand	ChiI		X	X	X	X	X	X	6	*R*
Government initiatives to inform and promote healthy lifestyle choices	GovH		X		X	X			3	*R*
Do you think foreign investors should be able to buy New Zealand farms?	IFF				X				1	1 = yes, 0 = no
Restricting foreign ownership of New Zealand farms	ForF						X	X	2	*R*
Restricting foreign ownership of New Zealand residential property	ForR						X	X	2	*R*
Increase payments for those receiving jobseeker support (formerly the Unemployment Benefit).	PayJ						X		1	
Increase payments for those receiving sole parent support (formerly the Domestic Purposes Benefit).	PayP						X		1	
Redistributing money and wealth more evenly among a larger percentage of the people in New Zealand through heavy taxes on the rich	IncR						X	X	2	*R*
Suppose a person has a painful incurable disease, do you think that doctors should be allowed by law to end the patient’s life if the patient requests it?	Euth						X		1	*R*
Ensuring that all food and food ingredients sold in New Zealand are free from genetically modified organisms	GMO						X	X	2	*R*
The current “3 Strikes” law for violent/sexual offenses, where the maximum possible sentence must be imposed without parole upon the third conviction	3stk						X		1	
A publicly available online database of all convicted sex offenders in New Zealand	SexO						X		1	
The New Zealand government should be involved in regulating carbon emissions	CarR	X	X	X	X	X	X		6	*R*
Increased government spending on new motorways	MtrS	X			X	X			3	*R*
Government subsidy of public transport	PubT	X			X	X			3	*R*
Protecting New Zealand’s native species should be a national priority	NatS		X			X			2	*R*
Do you support the use of 1080 poison for possum control in New Zealand?	PosC		X			X			2	*R*
Nodes per wave		23	28	26	33	37	40	31		

*Note*. With one exception (noted in table), all items are on a 1 to 7 scale. *R* = reverse score.

In 2011, New Zealand held an election to decide the nation’s next prime minister. We aimed to use measures of symbolic and operational components of belief systems from the wave taken before the election (Time 3) or the wave after the election (Time 4) and integrate them with measures of self-reported voting behavior, environmental behavior, and religious behavior taken after the election (Time 4). Using postelection voting behavior will presumably capture responses closer to their actual voting behavior as opposed to their anticipated voting behavior (see Sibley et al., 2017, on the correspondence between the NZAVS, other polling data, and election results). Voting behavior was measured by asking each participant who reported having voted in the most recent election to indicate the party for whom they voted. We included all of the possible parties that participants selected with the exception of Jim Anderton’s Progressive Party and the United Party (in the combined Wave 3 and 4 data), which were added to the Other category because of their low numbers. Participants who were unsure who they voted for (or who did not report who they voted for) were removed from the analyses using voting behavior. Environmental behavior was measured with two items that read, “Have you made sacrifices to your standard of living (e.g., accepted higher prices, driven less, conserved energy) in order to protect the environment?” and “Have you made changes to your daily routine in order to protect the environment?” Both were measured on a scale from 1 (*definitely no*) to 7 (*definitely yes*). Religious behavior was assessed by asking participants, “how many times did you attend a church or place of worship in the last month?” Any of the open-ended responses equal to or greater than 5 were recoded as 5.

## Results^1^

### What Is Most Central?

#### Network estimation

We estimate a network that consists of regularized partial correlation coefficients. The individual items were treated as nodes and the edges connecting them were estimated as regularized partial correlations, such that each edge represents the undirected association between each node while controlling for the influence of all of the other nodes in the network (Epskamp, & Fried, 2018; Lauritzen, 1996; for a tutorial, see Epskamp, Borsboom, & Fried, 2018). This modeling approach assumes that nodes in the belief system mutually influence each other and like to be in the same state as their neighbors (e.g., if two nodes are positively connected, they are likely to share the same liberal/conservative state). The first step in the network estimation procedure is to estimate a correlation matrix. For ordinal variables (variables with six or fewer integer values were treated as ordinal), polychoric correlations are estimated. This is the stage where missing data are estimated using full information maximum likelihood estimation. Then, the correlation matrix is inverted to create a partial correlation matrix and a LASSO (least absolute shrinkage and selection operator) regularization procedure, borrowed from machine learning, helps to control the possibility of spurious effects and shrinks small coefficients to zero (Costantini, Epskamp, et al., 2015; Epskamp & Fried, 2018). The networks for each wave were estimated using the qgraph and bootnet packages (default gamma tuning parameter = .5). The stability of the networks was estimated using the bootnet package (see Supplemental Materials).

An approach based on partial correlations, such as the one we are using here, is ideal for data that are continuous or quasi-continuous. This estimates a network that is linked by the unique associations between each of the nodes. The approach performs well in simulation studies (Schmittmann, Jahfari, Borsboom, Savi, & Waldorp, 2015; see also simulation in Supplemental Materials) and outperforms other similar methods (e.g., networks estimated using only correlations). The method we use is the most often used method to estimate network connections in a wide range of networks, including the networks that comprise individual attitudes (e.g., Dalege et al., 2015), personality traits (e.g., Costantini, Richetin, et al., 2015), and psychopathologies (e.g., Fried et al., 2018; see also Epskamp, Waldorp, Mõttus, & Borsboom, 2017; Epskamp & Fried, 2018; Foygel & Drton, 2010; Friedman, Hastie, & Tibshirani, 2008; Lauritzen, 1996; Meinshausen & Bühlmann, 2006).

Seven networks were estimated, one for each wave of available data (see Figures S1 and S2 in Supplemental Material). See Supplemental Materials for visualizations and robustness checks of the networks. We estimated the three types of centrality discussed above with methods for weighted networks (Opsahl et al., 2010) for each of the seven networks. Then, we tested to see if symbolic or operational components tended to have higher levels of centrality.

#### Hypothesis testing

**Figure 1.**
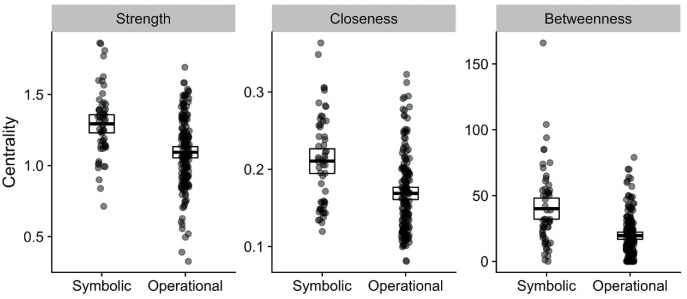
Symbolic components (n = 56) are higher in strength, closeness, and betweenness centrality than operational components (n = 162) across the seven belief system networks. Higher values imply higher centrality. Points represent individual nodes from networks estimated from all seven waves of data (visualized in Figures S1 and S2 in Supplemental Material). Points are horizontally jittered to improve clarify. Top and bottom edges of the boxes represent upper and lower bounds of the 95% confidence interval of the mean. The centerline of the box represents the mean. Closeness was multiplied by 100 before plotting to reduce the number of leading zeros.

Figure 1 plots the centrality estimates for each node of the seven networks visualized in Figures S1 and S2 in Supplemental Material. It shows that, overall, symbolic components were more central than operational components in terms of strength, *d* = 0.80, 95% CI = [0.49, 1.12], *F*(1, 204) = 35.01, *p* < .001; closeness, *d* = 0.78, 95% CI = [0.47, 1.09], *F*(1, 204) = 172.54, *p* < .001; and betweenness, *d* = 0.97, 95% CI = [0.65, 1.29], *F*(1, 204) = 40.19, *p* < .001 (results replicate using nonparametric methods). That is, consistent with the symbolic centric hypothesis, symbolic (vs. operational) components are more closely connected to their immediate neighbors in the network (strength), are more closely connected to all of the other nodes in the network (closeness), and help tie different nodes in the network together (betweenness). These findings replicated across waves (i.e., they were not moderated by the year of the survey). Notably, there is an overlap in the distribution of centrality estimates of symbolic and operational components. This suggests that individual operational components may have particularly high levels of centrality and that individual symbolic components may have particularly low levels of centrality, but that symbolic components are on average the most central. Although this is what one expects to find when analyzing nearly any kind of continuous variable, in this research domain it highlights that there is a range of centrality estimates and that the central versus peripheral distinction is a quantitative (not a qualitative) difference.

#### Additional checks

We conducted a number of additional checks on these results that are described in the Supplemental Materials. To ensure that the results were not due to the specific items available in any given wave, we repeated all analyses with items available in at least six of the seven waves. The results were the same. To check if the results were affected by the distribution of centrality scores across the waves, we ranked and standardized the centrality scores within each wave. Symbolic components were still the most central. To test if the networks differed between people with higher or lower levels of education and political knowledge, we estimated additional networks for these subgroups and compared the resulting networks. The relative centrality of symbolic and operational components did not differ based on political knowledge or education. This indicates that, while the belief systems of people from these groups do differ (for reviews see Converse, 2000; Federico & Malka, 2018), they do not differ in the extent to which symbolic components are more central than operational components.

### How Are Components Related to Behavior?

#### Network estimation

**Figure 2.**
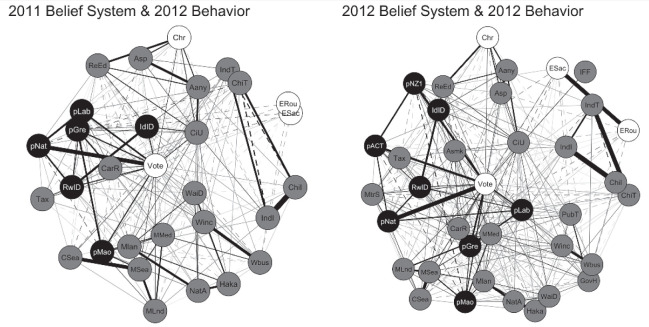
Belief system networks with symbolic (black nodes), operational (gray nodes), and behavioral (white nodes) components. Solid edges are positive. Dashed edges are negative. Thicker edges represent stronger connections between nodes. Placement of the nodes is determined with multidimensional scaling of the absolute value of the adjacency matrix (Jones, Mair, & McNally, 2018). Node labels are described in Table 1.

We estimated two additional networks that included nodes to represent voting and environmental behavior: one that combined the belief system data from Wave 3 (in 2011 before the election) with the behavior data from Wave 4 (in 2012 after the election; *n* = 4,190), and one with both belief system and behavior data from Wave 4 (after the election; *n* = 5,829). We estimated regularized partial correlation networks and treated voting behavior as a categorical variable with more than two categories, similar to a multinomial regression analysis (Haslbeck & Waldorp, 2016). The edges between the nodes and voting behavior thus reflect the absolute value of the average effect of the nodes on voting behavior across the categories (i.e., how much a node is associated with participants’ voting decision, regardless of the particular party). The edges between the remaining nodes reflect partial correlations. To measure the closeness of behavior to the nodes in the network, for each node of the two behavior networks, we calculated the distance of its shortest path between the node and each of the behavioral nodes using Dijkstra’s (1959) algorithm. Shorter paths are closer in the network. Figure 2 displays these networks.

**Figure 3.**
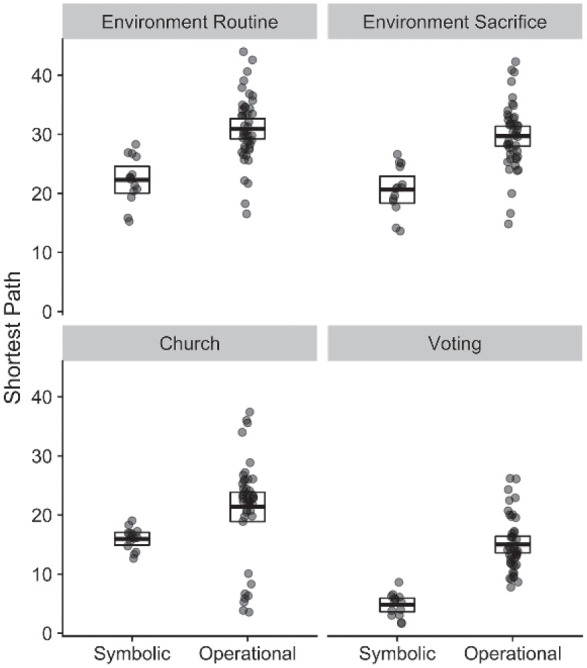
Symbolic components (*n* = 14) are more closely connected (have shorter paths) with behaviors than operational components (*n* = 45). Lower values imply higher centrality. Points represent individual nodes from both panels of Figure 2. Points are horizontally jittered to improve clarity. Top and bottom edges of the boxes represent upper and lower bounds of the 95% confidence interval of the mean. The centerline of the box represents the mean.

For each of the four behavioral nodes, we tested whether symbolic components or operational components had shorter paths to the behavioral nodes. The lower the value, the more central (i.e., close to behaviors) the component. Accordingly, Figure 3 shows that symbolic components of the belief system were closer than operational components to voting behavior, *d* = −2.43, 95% CI = [–3.19, −1.67], *F*(1, 55) = 61.21, *p* < .001. Environmental and religious behaviors are not as close to the belief system as a whole (perhaps reflecting their less clear connection with politics compared to voting behavior). However, the difference between symbolic and operational components was also found for changes in routines, *d* = −1.62, 95% CI = [−2.31, −0.95], *F*(1, 55) = 27.45, *p* < .001, and sacrifices made, *d* = −1.71, 95% CI = [−2.40, −1.02], *F*(1, 55) = 30.32, *p* < .001, that benefit the environment, as well as religious behavior, *d* = −0.74, 95% CI = [−1.37, −0.11], *F*(1, 55) = 5.92, *p* < .001. These differences replicated across the 2 years (i.e., the results were not moderated by year) and were robust to the inclusion and exclusion of different nodes in the network, as well as across levels of education and political knowledge (see Supplemental Materials for full results). Notably, there is an overlap in the distribution of shortest paths, suggesting that individual operational components may have particularly close associations with political behavior. Nevertheless, symbolic components are on average the most central.

## Discussion

By combining network psychometrics with research on operational and symbolic components of belief systems, we were able to model the belief system in a way that matches theoretical definitions of the construct while using a large number of elements of the political belief system. This allowed us to examine how different types of nodes are situated within the broad belief system. We found, and replicated across multiple years, that symbolic components of the belief system were more central than operational components to the overall system and were closer to multiple types of politically relevant behavior. That is, when it comes to political belief systems, our symbolic attachments to parties and labels are a more important part of the belief system than the actual policy positions. This echoes work that has used less comprehensive methods for probing the deep structure of political belief systems (Converse, 1964; Kinder & Kalmoe, 2017; Malka & Lelkes, 2010) and those that have used other variations of the network approach (Boutyline & Vaisey, 2017).

### Other Explanations for Variation in Centrality?

Notably, not all operational components are created equally and they are not all equally peripheral to the belief system. Indeed, some operational components are substantially more central than the average symbolic component. This suggests that some operational components can serve as relatively more central components to the belief system, at least in some situations. For example, across the 7 years, the operational item about teaching Māori (i.e., the indigenous peoples of New Zealand) language in public school (node “Mlan” in Figure 2) was in the top 73% to 96% of nodes for strength centrality, the top 66% to 78% of nodes for closeness centrality, and the top 71% to 97% of nodes for betweenness centrality, suggesting that, overall, it was a relatively central component. One possible explanation is that this item taps into other types of group attachments (e.g., ethnic group) that are relevant for politics in New Zealand, contaminating this operational node with symbolic content (e.g., a type of symbolic racism; Sears & Henry, 2005). And indeed, such arguments have been made for why some attitudes are more closely connected to ideological or partisan identification in the United States than are other attitudes (e.g., Converse, 1964; Kinder & Kalmoe, 2017).

However, other operational items are also related to group attachments to some degree and these items are not consistently central (e.g., issues regarding immigration with India and China, or the role of women in the workforce). One possibility is that it is not just group attachments *per se* that account for this variability, but the extent an issue is “hard” (i.e., requires deliberation, is complex) or “easy” (i.e., comes from the “gut,” more symbolic than technical; Carmines & Stimson, 1980). This is also unlikely to be the whole story as many issues likely to be “easy” are not particularly central (e.g., the aforementioned trade and immigration items, an item about performing the Haka, etc.).

To more formally test this idea, we divided the items into items tapping into social policies and those tapping economic policies (a few items were ambiguous and were not included here). Our assumption is that, on average, social policies represent easy issues and economic policies represent hard issues. When centrality of these two subtypes are compared, we only find significant differences for strength centrality (*p* = .04), where social policies are slightly more strength central than economic policies. Although this is consistent with the idea that easy issues will be more central, it is far from conclusive.

Just as some operational components are particularly central, there is substantial variation in the centrality of the symbolic components. One distinction that has occupied scholars is the relative centrality of partisan versus ideological forms of symbolic components. That is, is party support/identification or ideological identification more central? The most current evidence suggests that party identity is more central than ideological identity (Kinder & Kalmoe, 2017; see also Green et al., 2002). To formally test this idea in our data, we divided the items into those tapping into party support/identity and those tapping into ideological identification. A clear limitation of this analysis is the low number of items assessing ideological identification (only 1 or 2 per year). With this caveat in mind, we only find significant differences between party support/identity and ideology for strength centrality (*p* = .03) in that the partisan support components are slightly more strength central than ideological identification. Although these results are consistent with the idea that party identity will be more central, it is again far from conclusive.

Understanding what is associated with network centrality beyond symbolic and operational categories will be an important task for future work. By allowing us to test what type of component is most central and to quantify the centrality of all of the individual nodes, our approach allows for a more continuous understanding of belief system centrality than the typical approaches used in the literature, which tend to encourage dichotomous categorizations of central versus peripheral components.

### Limitations and Future Directions

Our approach does have its limitations. By conducting cross-sectional analyses, the edges of the networks have no clear causal meaning. Although they represent potential causal links between nodes, the analyses have all of the same shortcomings as any cross-sectional analysis. Nevertheless, the support we find for the symbolic centric hypothesis is consistent with experimental work showing that policy issues extend from our party and ideological attachments (i.e., symbolic operational) and so serves as a complement, rather than a replacement, to experimental approaches that focus on different subsections of the belief system (e.g., Huddy et al., 2015; Kahan et al., 2017; Malka & Lelkes, 2010). This limitation does raise the question of what is centrality without the causal component (i.e., what is centrality in this study)? There are two responses to this question. First, it may be possible that some components with high centrality are never the cause and only the consequence of other components. Although we find this possibility unlikely, it is still be illuminating to know if there is a component of the belief system that the other components of the system cause. It would suggest the focus of that particular belief system. However, this is not our favored answer. Rather, a second response to this question is that there *is* causal information gathered from a number of rigorous experimental and quasi-experimental studies throughout the years (and cited throughout). When those conclusions are combined with the current data, we know both where symbolic and operational components are situated in the system as a whole (our data) and what the causal direction is (experimental work). Both types of data are needed to make convincing claims of centrality.

Our cross-sectional analyses also mean that the networks we estimate are primarily applicable for describing between-person differences and similarities in attitudes and beliefs (Epskamp et al., 2017). That is, these belief system networks are not a snapshot inside of any one person’s head, but rather, they capture a summary of the attitudinal and belief-based cleavages between people. Future studies, with intensive longitudinal data (e.g., over 40 time points), may be able to take advantage of additional methods that can estimate individualized networks that provide snapshots of a given person’s belief system and how it evolves over time (cf. Bringmann et al., 2013).

The findings provide guidance for people and politicians who want to attach new issues to a political belief system. By linking new issues with symbolic components, such as party attachments or other political labels, it should be easier to embed the new issue within the broader network of attitudes and beliefs. For the researcher, our results also suggest that symbolic components of the belief system are key to predicting where people will fall on new issues or politically relevant behaviors, whereas issue-based measures are less relevant (cf. Dalege, Borsboom, Harreveld, Waldorp, & van der Maas, 2017). Going further, the belief system networks we estimate might be used to identify which issues may be more amenable to change by entrepreneurial politicians and parties (e.g., De Vries & Hobolt, 2012) because they are relatively loosely connected to the rest of the belief system.

Our methodological approach can also open other new doors. For example, although the belief systems we estimated have a large number of connections between components, many of these connections are weak. However, it should be possible to identify the most constrained and interrelated subsections of the belief system to identify “small ideologies” that are the topics where people will have the most consistent, stable, and interconnected opinions. And then to use these belief system networks and their various subcomponents to dynamically model how belief systems are expected to change over time, or when facing social pressures (Friedkin, Proskurnikov, Tempo, & Parsegov, 2016).

Overall, by modeling belief systems as networks, we are able to locate specific attitudes and beliefs within a larger and more complete belief system. In doing so, we demonstrate that symbolic components are more central to the belief system and are closer to politically relevant behavior than are operational components. Such a network approach provides a modeling approach that matches the conceptual definition of belief systems and opens the door to new research at the intersection of belief systems research and network science.

## Supplemental Material

beliefsystemcentrality.som – Supplemental material for What Is Central to Political Belief System Networks? Supplemental material, beliefsystemcentrality.som for What Is Central to Political Belief System Networks? by Mark J. Brandt, Chris G. Sibley and Danny Osborne in Personality and Social Psychology Bulletin

Brandt_Online_Appendix – Supplemental material for What Is Central to Political Belief System Networks? Supplemental material, Brandt_Online_Appendix for What Is Central to Political Belief System Networks? by Mark J. Brandt, Chris G. Sibley and Danny Osborne in Personality and Social Psychology Bulletin

PSPB824354_Supplemental_Material_CLN – Supplemental material for What Is Central to Political Belief System Networks? Supplemental material, PSPB824354_Supplemental_Material_CLN for What Is Central to Political Belief System Networks? by Mark J. Brandt, Chris G. Sibley and Danny Osborne in Personality and Social Psychology Bulletin

## Notes

1. We used R Version 3.4.2 (2017) with bootnet (Epskamp, Borsboom, & Fried, 2018), cowplot (Wilke, 2017), tidyverse (Wickham, 2017), mgm (Haslbeck & Waldorp, 2016), NetworkComparisonTest (van Borkulo, Epskamp, & Millner, 2016), qgraph (Epskamp, Cramer, Waldorp, Schmittmann, & Borsboom, 2012), rio (Chan, Chan, Leeper, & Becker, 2016), psych (Revelle, 2017), tidystats (Sleeger, 2017), and effesize (Torchiano, 2017), networktools (Jones, 2018), and reshape2 (Wickham, 2007) for all our analyses and figures throughout the article.
