# Supplementary material for: What Is Central to Political Belief System Networks?
Source: Pers Soc Psychol Bull. 2019 Jan 28;45(9):1352–64. doi: 10.1177/0146167218824354 (PMC6676336; doi:10.1177/0146167218824354)
Supplement: beliefsystemcentrality.som – Supplemental material for What Is Central to Political Belief System Networks? [file beliefsystemcentrality.som.pdf]

# Supplemental Materials

## Table of Contents

Supplemental Materials .......... 1
Table of Contents .......... 1
Summary of Political Situation in New Zealand .......... 1
Network Stability .......... 2
Centrality Stability .......... 2
Simulated Specificity, Sensitivity, and Stability .......... 3
Complete Results from the Main Text .......... 6
Standardizing Centrality Estimates .......... 6
Robustness Checks on the Inclusion of Nodes .......... 6
Political Knowledge & Education Analyses .......... 7
Political Knowledge Results .......... 8
Education Results .......... 9
Different Subtypes of Symbolic and Operational Components .......... 9
Figures .......... 11
Tables .......... 38
Supplemental References .......... 49

## Summary of Political Situation in New Zealand

Given that our sample comes from a relatively under-studied national context, a (brief) discussion of New Zealand politics is needed. New Zealand is a small (i.e., as of June 2018, roughly 4.9 million people; Statistics New Zealand, 2018) democratic nation in the South Pacific with a Mixed-Member Proportional (MMP) electoral system. Instead of first-past-the-post systems whereby the party with the most votes wins the given seat (as in the United States), MMP gives voters two votes: One vote for a political party (a party vote), and one vote for a local representative (an electorate vote). Parties that receive 5% or more of the national party vote and/or the plurality of an electorate vote are allocated a seat in Parliament (see Electoral Commission, 2018). As such, New Zealand Parliament consists of a relatively large number of parties (i.e., between seven and eight parties had one or more seats in Parliament during the first seven waves of the NZAVS), including Labour (the main centre-left party) and National (the main centre-right party), as well as various minor parties from the left (e.g., the Green Party, the Māori Party, and the Mana Party), right (e.g., the ACT Party and the Conservative Party), and centre (e.g., United Future). Nevertheless, left vs.

right (or liberal vs. conservative) cleavages are still present whereby liberal/left-wing parties tend to support progressive social and economic policies more than do the conservative/right-wing parties (although it is worth noting that the National Party, which tends to be centre-right formed a coalitional government with the Māori Party, which represents Indigenous rights, during two of the three recent consecutive three-year terms they held power; i.e., 2008-2014).

Despite having some familiar political cleavages, New Zealand is a post-colonial—and formally bicultural—society characterized by a precarious relationship between Māori (i.e., the indigenous peoples of New Zealand) and New Zealand Europeans (i.e., descendants of British colonizers). Although the British colonizers formally signed a treaty with Māori chiefs in 1840 (namely, Te Tiriti o Waitangi) that granted Māori the same rights as the British settlers (as well as sovereignty over their possessions), British colonizers soon engaged in a systematic confiscation of Māori land and property, leaving the indigenous peoples of New Zealand with a legacy of injustices that persist today (see Orange, 1992). As such, much of contemporary New Zealand politics revolves around the denial (vs. recognition) of the relevance of these past injustices to contemporary society, as well as the extent to which Māori culture should be incorporated into the nation's identity (see Sibley & Osborne, 2016). These culture-specific ideologies are highly influential in New Zealand politics and correlate with a number of socio-political outcomes including political party preferences (Greaves, Osborne, Sengupta, Milojev, & Sibley, 2014), resource-based policy preferences (Newton, Sibley, & Osborne, 2018), and support for collective action (Osborne, Yogeeswaran, & Sibley, 2017).

**Network Stability**

Throughout the paper, we determined our sample size by using all of the available data given the variables used in the network. The typical way of thinking about statistical power does not apply well to networks because they are not estimated using null hypothesis significance testing, but instead utilize a modified LASSO regression procedure. However, it is possible to estimate the precision of network edges (Epskamp, Borsboom, & Fried, 2017). The precision of the edges and their bootstrapped 95%CI are Figures S3-S9. All of the edges, across the networks, are precisely estimated.

**Centrality Stability**

All of our primary analyses are based on indicators of centrality. If these estimates are not stable, then none of our results are likely to be stable. To test for the stability of these estimates we used the boostrapping procedure in bootnet that re-estimates the network for different subsets of cases. Then, the centrality estimates from the original network and these re-estimated networks are correlated. Espskamp et al (2017) recommend that this correlation should be at least .75 when 25% of the sample is dropped. In all cases, we are able to drop substantially more cases (~75%) and retain a correlation of at least .75 between the three centrality estimates for the original and re-estimated networks. See Table S2 for these estimates. This shows that the networks used in our analysis are highly stable.

## Simulated Specificity, Sensitivity, and Stability

As an additional check on the ability of our data to estimate the networks, we used the network from Wave 6 (our largest network) to simulate the specificity (true-negative) and sensitivity (true-positive) rates for edges in the networks at varying sample sizes (Epskamp & Fried, in press). We used sample sizes of 500 and 1000 as base comparisons and then additional sample sizes based on the sample sizes we used in our own study. In all cases, the networks we estimated had acceptable levels of both parameters, although the networks with smaller sample sizes have lower selectivity (Figure S10). We can also correlate the edge weights from simulated networks at varying sample sizes to a "true" network (Figure S10), as well as the centrality estimates from simulated and true networks (Figure S11; Epskamp & Fried, in press). These analyses are crucial when considering the overall structure of the networks and for when centrality estimates are of interest (as they are in our case). In all cases, the correlations are high given our sample sizes.

## Simulation Study

Regularized partial correlation networks, like the ones used in our study, are new to political psychology and political science. Therefore, we conducted a simulation study to understand how well the regularized partial correlation method that we used (EBICglasso) compared to methods using non-regularized partial correlations or correlations. We wanted to know if correlation networks, partial correlation networks, or regularized partial correlation networks with EBICglasso were most effective at recovering network structure. To test this, we simulated eight different weighted networks consisting of 10 nodes and random connectivity. Four networks had higher levels of connectivity (proportion of links = .6) and

four networks had lower levels of connectivity (proportion of links = .3). The networks were constructed using the genGGM function of the bootnet package (Epskamp, Borsboom, & Fried, 2017), which generates a random network based on Erdos and Reyni (1959) and then constructs a weighted network using the approach of Yin and Li (2011). The networks were specified to have 75% positive edges. These eight networks served as input graphs into the simulation.

The input networks that serve as our "true" networks are in Figures S12 and S13. The maximum edge across the four high connectivity networks is 0.24, the minimum edge across the four networks is -0.30, the mean edge across the four networks is 0.03, and the proportion of edges with a non-zero weight is 0.64. The maximum edge across the four high connectivity networks is 0.53, the minimum edge across the four networks is -0.37, the mean edge across the four networks is 0.02, and the proportion of edges with a non-zero weight is 0.32. For each network, we simulated 25 data sets with 500, 1000, 2000, and 5000 cases. We simulated ordinal data with 7 levels. The simulation was conducted using the netSimulator function of the bootnet package.

We focus on five indicators of estimator performance. *Bias* is the absolute value of the difference between the edges in the true network and the estimated network. *Correlation* is the correlation between the edges in the estimated networks to the edges in the true network. *Betweenness*, *Closeness*, and *Strength* are the correlations between the centrality estimates in the estimated networks to the centrality estimates in the true network. A figure with the error rates and the correlations between the edges is in Figure S14. A figure with the correlations between centrality estimates are in Figure S15.

**Lower Connectivity**. When looking at the bias in edge weights (Figure S14) for the lower connectivity networks, the EBICglasso method had the lowest bias compared to the other two methods and the correlation method had the highest levels of bias across all of the sample sizes we tested. Similarly, when looking at the correlation between edge weights in the true and the simulated networks (Figure S14), we see that the EBICglasso has the highest correlation. Although the other two methods also had relatively high correlations between true and simulated edge weights, they were typically lower than the EBICglasso method.

When looking at the correlations between the centrality measure for the true network compared to the simulated networks (Figure S15), we find that all three methods perform

similarly for betweenness and closeness centrality, although both the EBICglasso and the partial correlation methods perform better than the correlation method at the highest sample size. For strength centrality, the EBICglasso and partial correlation methods perform better than the correlation method at all sample sizes.

**Higher Connectivity**. When looking at the bias in edge weights (Figure S14) for the higher connectivity networks, the EBICglasso method had the highest bias compared to the other two methods at the lowest sample size. As sample size increased, the bias for the EBICglasso decreased faster than it did for either of the other two methods, so that at the highest sample sizes EBICglasso and partial correlation methods had similar levels of bias and were more accurate than the correlation method. Similarly, when looking at the correlation between edge weights in the true and the simulated networks (Figure S14), we see that the EBICglasso method had the lowest correlation compared to the other two methods at the lowest sample size. As sample size increased, the correlation for the EBICglasso increased faster than it did for either of the other two methods, so that at the highest sample sizes EBICglasso and partial correlation methods had similar levels of correlations between the edges in the true and simulated networks and were more accurate than the correlation method.

When looking at the correlations between the centrality measures for the true network compared to the simulated networks (Figure S15), we find that the best method depends on the sample size. For betweenness, at the smallest sample size, both the correlation and partial correlation methods perform better than the EBICglasso. However, EBICglasso performs similarly well at the highest sample sizes. For closeness, at the smallest sample size, both the correlation and the partial correlation method perform better than the EBICglasso. However, at higher sample sizes the EBICglasso performs similarly well. At the highest sample sizes, the EBICglasso and partial correlation methods perform better than the correlation method. For strength, at the smallest sample size, both the correlation and partial correlation methods perform better than the EBICglasso. At higher sample sizes, the EBICglasso and partial correlation methods perform better than the correlation method

**Simulation Conclusion**. In our paper, we are using sample sizes that are greater than 4,000 people. When looking at the simulation results for the higher sample sizes (i.e. N = 2,000 or 5,000) the EBICglasso method typically performs better than the correlation method and it forms equally well, for higher connectivity networks, as the partial correlation network.

That is, in the type of data we are using in the current study the EBICglasso appears to be the method that is most likely to return the most accurate estimation of the underlying true network.

## Complete Results from the Main Text

The main text reported the main effects of the type of component. Here we report the complete ANOVAs in Table S3 and S4.

## Standardizing Centrality Estimates

Centrality estimates can be influenced by the edges (i.e. paths) between nodes of the network, but also by the overall size of the network. For example, if a network has more nodes, each individual nodes has more chances to be on the shortest path between two other nodes and so will have greater chance of receiving a high betweenness score. To ensure that our conclusions are robust to this potential challenge, within each wave we ranked the centrality estimates and then standardized these ranks to range from 0 to 1 (see also Boutyline & Vaisy, 2017). We then re-tested whether symbolic or operational components are more central and if this differed by year (note that the standardization within wave means that there will not be a main effect of wave, but the interaction between wave and type of component still gives us information if the effects differ by year). Results are reported in Table S5 and visualized in Figure S16.

## Robustness Checks on the Inclusion of Nodes

Network structure is determined, in part, by the inclusion of particular nodes. If a node is added or removed it changes the structure of the network. Although it is not possible to test the hypothesis that we have included all of the nodes that represent all of the components of the New Zealand political belief system, we can see if our overall conclusions hold when adding or removing nodes from the network. The results we already report speak against the addition of nodes as a threat to the validity of our findings: each wave of the study included different numbers of nodes and the specific numbers of nodes did not affect the relative centrality of symbolic or operational components of the belief system (there was not interaction between year of survey and the type of component). We also re-estimated all of our main findings only using items included in at least 6 of the 7 waves. The centrality results for strength were in the same direction as in the main text, but were not significant at the $p <$

.05 level. The results for centrality and betweenness are clearly consistent with those reported in the main text. The shortest path analyses replicated those in the main text. See Table S6 and S7 for results and Figure S18 and S19 for a visualization of these results.

**Political Knowledge & Education Analyses**

We tested if our results differed for people with high levels of political knowledge or education compared to people with low levels of political knowledge or education. Past work suggests that people with higher levels of political knowledge and education are more likely to think in ideological terms and have differently structured belief systems (Converse, 1964; Federico & Schneider, 2007; Zaller, 1992). We measured both of these constructs and tested if the results were similar or different across them.

The NZAVS included an online-only survey wave between Wave 3 and Wave 4 which included a seven-item measure/quiz of political knowledge (e.g., who is the Minister of Finance). Participants selected an answer from 2 to 8 answer options, with the addition of an option to indicate that they did not know the answer. Responses were coded as correct or incorrect and answers of "don't know" were coded as incorrect. We included participants who completed this measure and the items from the other waves. Using the item response options of the psych package (Revelle, 2017), we calculated each participant's level of political knowledge, recognizing that some of the factual questions might be more difficult to answer than other questions. Then, we estimated networks for people high and low in political knowledge. Although it is ideal to treat continuous variables as continuous (see MacCallum, Zhang, Preacher, & Rucker, 2002), that is not possible with this approach (cf. Isvoranu, Borsboom, Os, & Guloksuz, 2016). Therefore, we conducted a median split and estimated networks for each of the seven waves for participants who fell into each of these two groups (Low Knowledge: 2009 Wave n = 960, 2010 Wave n = 883, 2011 Wave n = 1688, 2012 Wave n = 1651, 2013 Wave n = 1582, Wave 2014 n = 1504, Wave 2015 n = 1370; High Knowledge: 2009 Wave n = 780, 2010 Wave n = 721, 2011 Wave n = 1866, 2012 Wave n = 1827, 2013 Wave n = 1776, Wave 2014 n = 1712, Wave 2015 n = 1597). Network estimation was completed using the same techniques described above.

Participants self-reported their education in the 2009, 2012, 2013, 2014, 2015 Waves. We categorized people into two groups: people who completed post-secondary education (High Education: 2009 Wave n = 1642, 2010 Wave n = 1187, 2011 Wave n = 2344, 2012

Wave n = 4517, 2013 Wave n = 7273, Wave 2014 n = 6652, Wave 2015 n = 6004) and those who did not (Low Education: 2009 Wave n = 3642, 2010 Wave n = 2480, 2011 Wave n = 3002, 2012 Wave n = 6499, 2013 Wave n = 9413, Wave 2014 n = 7956, Wave 2015 n = 7511) and estimated networks for each group. When education was not assessed in a wave, we used participants' education level from the nearest available wave (e.g., 2009 Wave education for estimating 2010 Wave network). Network estimation was completed using the same techniques described above.

**Political Knowledge Results**

The political knowledge networks are in Figures S20 – S23. In addition to the networks for people scoring high and low on political knowledge, we also plotted a difference network that shows the edges of the two networks that differ by |.10| or more. We first compared the overall strength of high and low knowledge networks using a permutation test method implemented in the NetworkComparisonTest package (van Borkulo et al., 2016). This analysis computes a statistic S that is the difference in the global strengths between the two networks that are compared. It revealed only one difference (Table S8). In the 2014 Wave the global strength of the network was stronger for people with high political knowledge than for people low in political knowledge.

To test if the results reported in the main text were similar for people with both high and low levels of political knowledge, we also computed centrality statistics for each of the nodes in the high and low knowledge networks. Then we tested to see if operational and symbolic nodes were more or less central and if this differed by year or level of political knowledge. This analyses did not reveal any consistent effects for political knowledge. This is not to say that there were no significant effects (see Figure S24 and Tables S9 – S11). Rather, the effects are not consistent across measures. For strength, there is a nearly non-significant effect of political knowledge, such that components in high knowledge networks are somewhat stronger than components in low knowledge networks. For closeness, political knowledge significantly interacts with wave. In some waves, high knowledge networks had more closeness than low knowledge networks, but in other waves there was no difference, or even reversed. For betweenness, political knowledge significantly interacts with whether the item is symbolic or operational. As is clear in Figure S24, symbolic components are more central for low political knowledge networks than for high political knowledge networks, but there is no clear difference for operational components. Because there were not clear and

consistent effects of political knowledge and because the finding that symbolic components are more central than operational components were robust across levels of political knowledge and waves, we focus on this finding and put aside the potential differences by political knowledge for future work.

After reading these results, a reviewer requested that we focus on people in the upper and lower quartiles of political knowledge. Therefore, we repeated the analyses above with this more limited sample. The conclusions do not change. See Figure S25 and Tables S12 to S14.

After reading these results, a different reviewer requested that we examine whether political knowledge affects the behavioral results. We re-ran all of the behavioral analyses separately for people with high and low levels of political knowledge (defined by the median split described above). See Figure S26 and Table S15 for results. Behaviors were closer to symbolic than operational components for people with both high and low levels of political knowledge. Conceptually similar analyses were conducted for people high and low in education (see Figure 27 and Table S16). Again, behaviors were closer to symbolic than operational components for people with both high and low levels of education.

**Education Results**

The education networks are in Figures S28 – S31. In addition to the networks for people with high and low levels of education, we also plotted a difference network that shows the edges of the two networks that differ by |.10| or more. We first compared the overall strength of the high and low education networks using a permutation test method implemented in the NetworkComparisonTest package (van Borkulo et al., 2016). This analysis revealed no differences in overall strength of the high and low education level networks (Table S8).

We also computed centrality statistics for each of the nodes in the high and low education networks. Then we tested to see if operational and symbolic nodes were more or less central and if this differed by level of education. This analyses did not reveal any consistent effects for education. See Figure S31 and Tables S17 – S19.

## Different Subtypes of Symbolic and Operational Components

After seeing these results, two reviewers asked if different types of symbolic and operational components accounted for some of the variation in centrality. That is, some work has found that partisan identification shows signs of higher centrality than does ideological identification (citations) and this distinction might account for variation in centrality among symbolic components. Similarly, some work has found that social policies show signs of higher centrality than do economic policies (citations) and this distinction might account for variation in centrality among operational components. To test this, we separately categorized the party support items and the ideological identification items (i.e. both types of symbolic items) and we separately categorized social policies and economic policies (i.e. both types of operational items). Some policies were not easily categorized and were removed from these analyses. The ANOVAs testing these results are in Table S20 and visualized in Figure S33. Pairwise comparisons between the four subtypes are in Table S21. In all cases, there are significant main effects of subtype. These main effects were not moderated by wave.

The key follow-up comparisons are between the subtypes within the symbolic and operational components. It does not appear that these distinctions account for variation with the specific components. Specifically, ideological and partisan support were not significantly different in terms of closeness and betweenness; however, there was a statistically significant difference in terms of strength. Similarly, economic and social policy support were not significantly different in terms of closeness and betweenness; however, there was a statistically significant difference in terms of strength.

## Figures

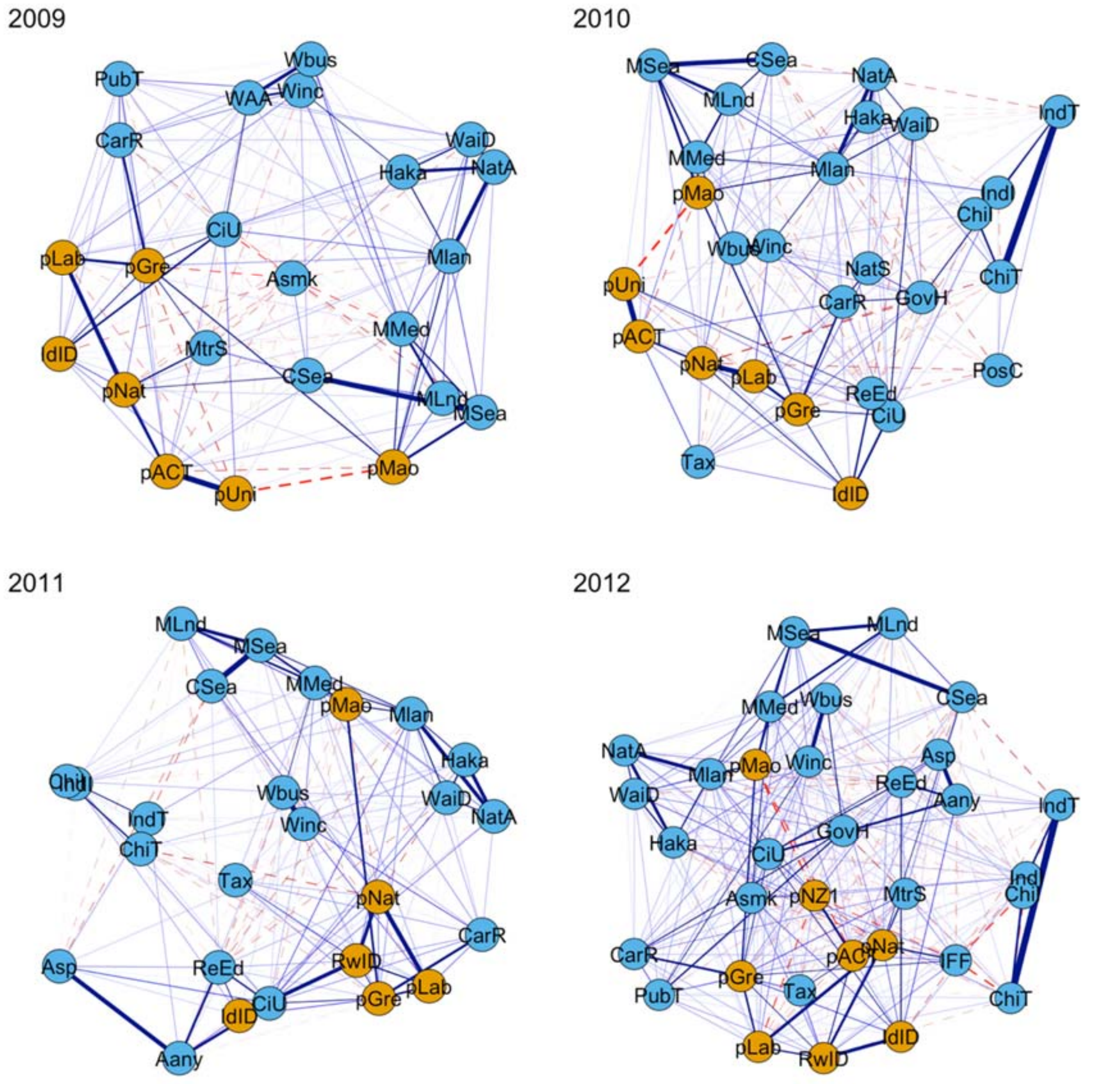


**Figure S1. Belief system networks with symbolic (gold nodes) and operational (blue nodes) components for Wave 1 – Wave 4. Solid blue edges are positive. Dashed red edges are negative. Thicker edges represent stronger connections between nodes. Placement of the nodes is determined with multidimensional scaling of the absolute value of the adjacency matrix (Jones, Mair, & McNally, 2018).**

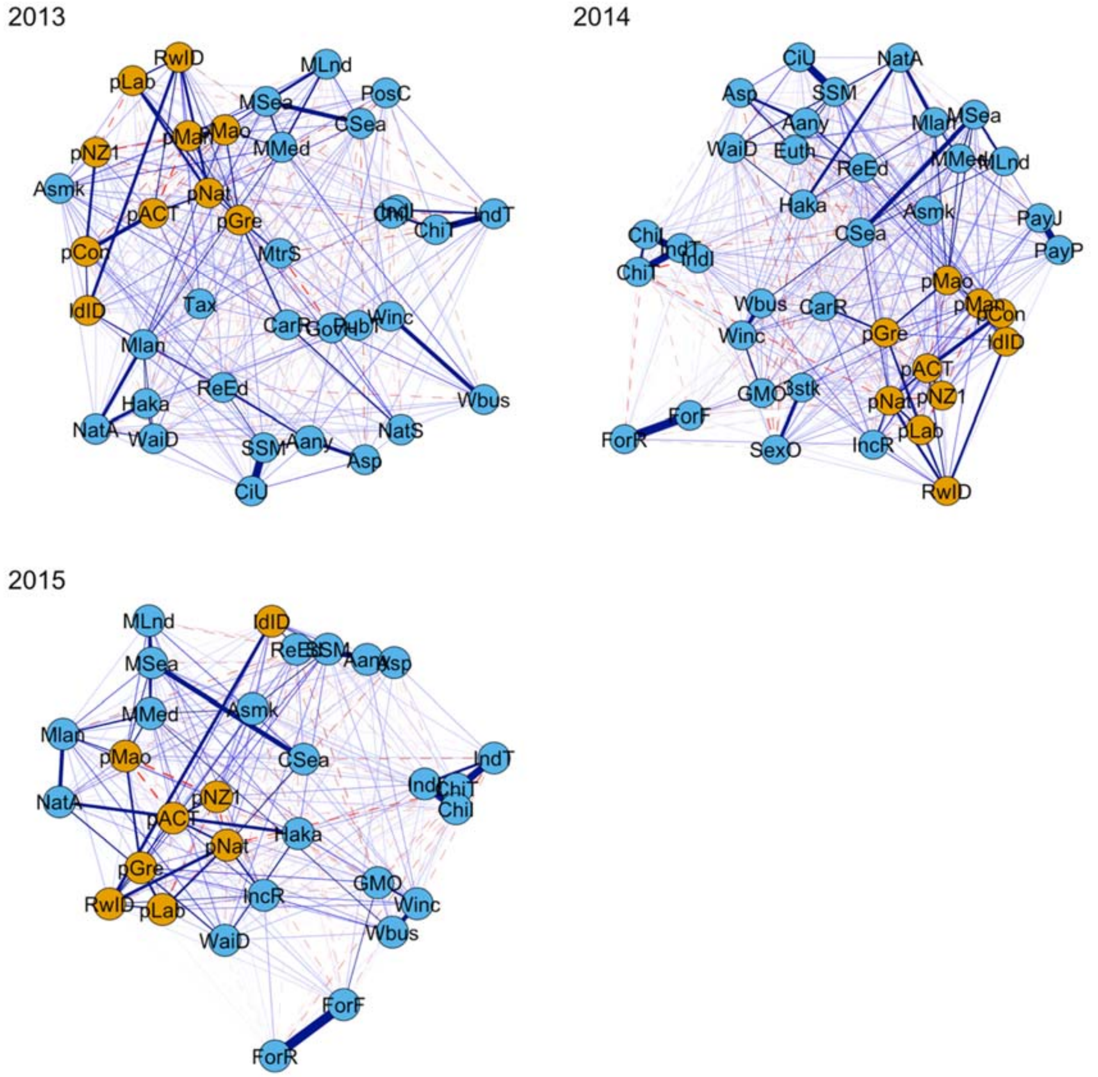


**Figure S2. Belief system networks with symbolic (gold nodes) and operational (blue nodes) components for Wave 5 – Wave 7. Solid blue edges are positive. Dashed red edges are negative. Thicker edges represent stronger connections between nodes. Placement of the nodes is determined with multidimensional scaling of the absolute value of the adjacency matrix (Jones, Mair, & McNally, 2018).**

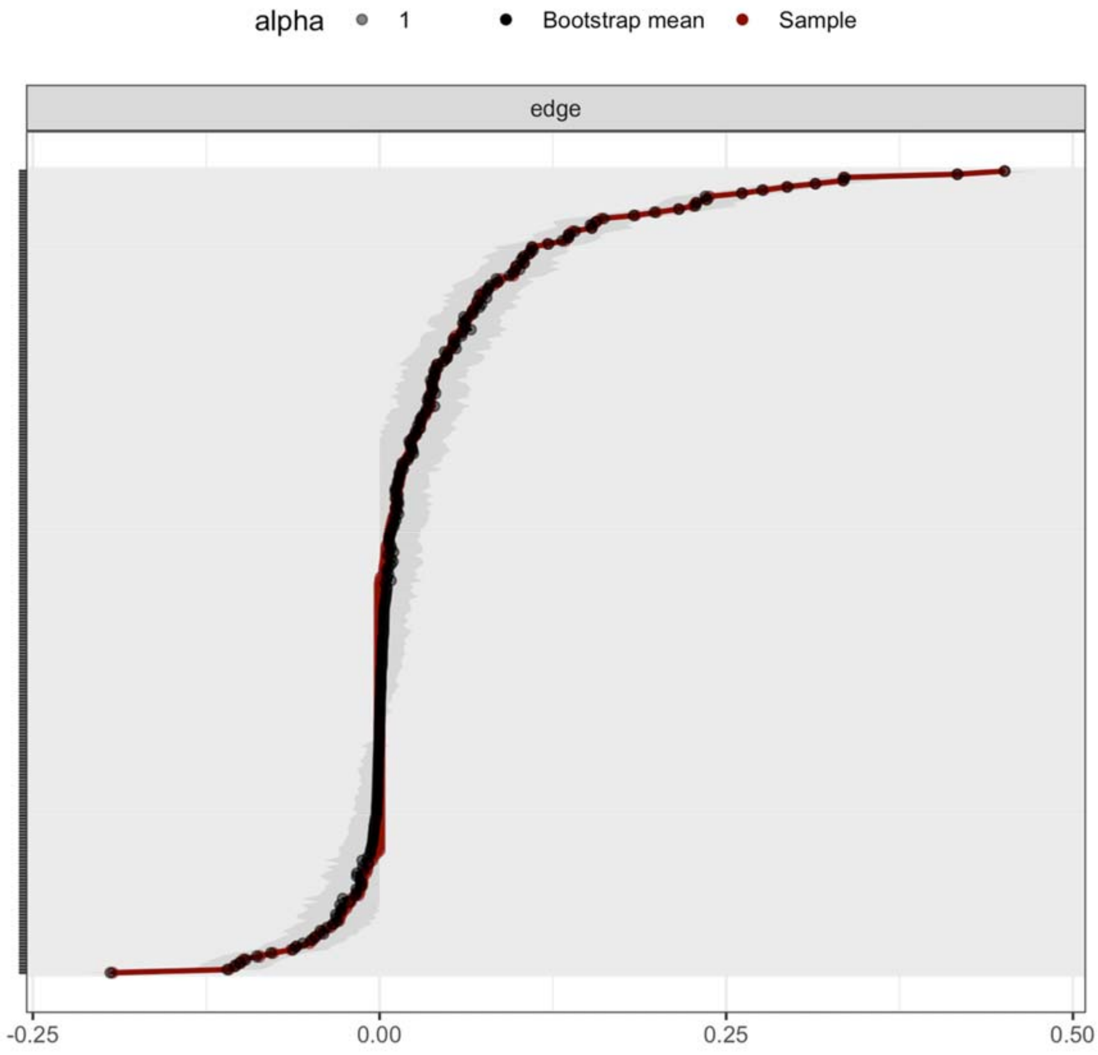


**Figure S3. Edge estimates (red dots) with bootstrapped 95% confidence interval (gray shaded region) for Wave 1. Each dot represents an edge. Edge labels are suppressed to maintain legibility.**

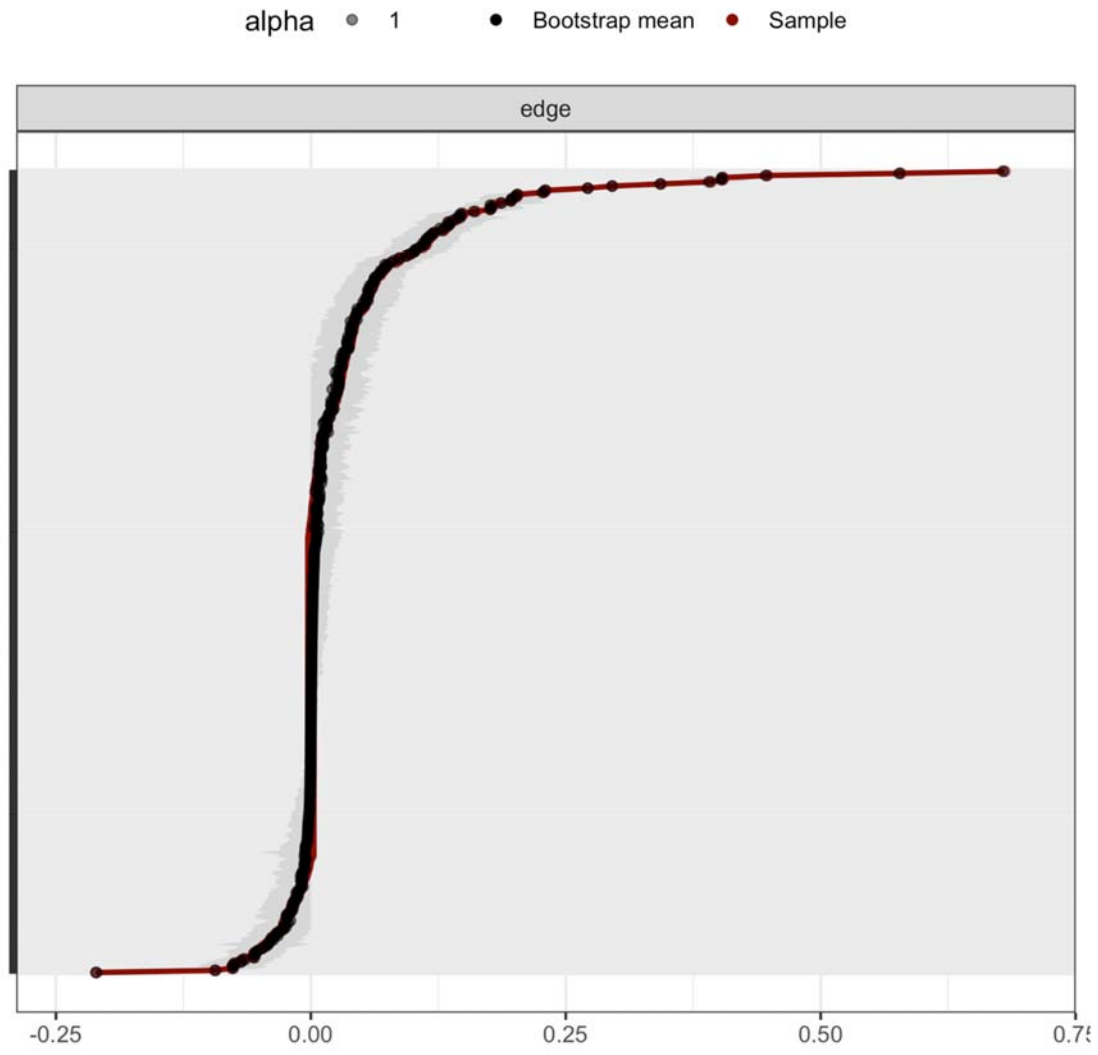


**Figure S4. Edge estimates (red dots) with bootstrapped 95% confidence interval (gray shaded region) for Wave 2. Each dot represents an edge. Edge labels are suppressed to maintain legibility.**

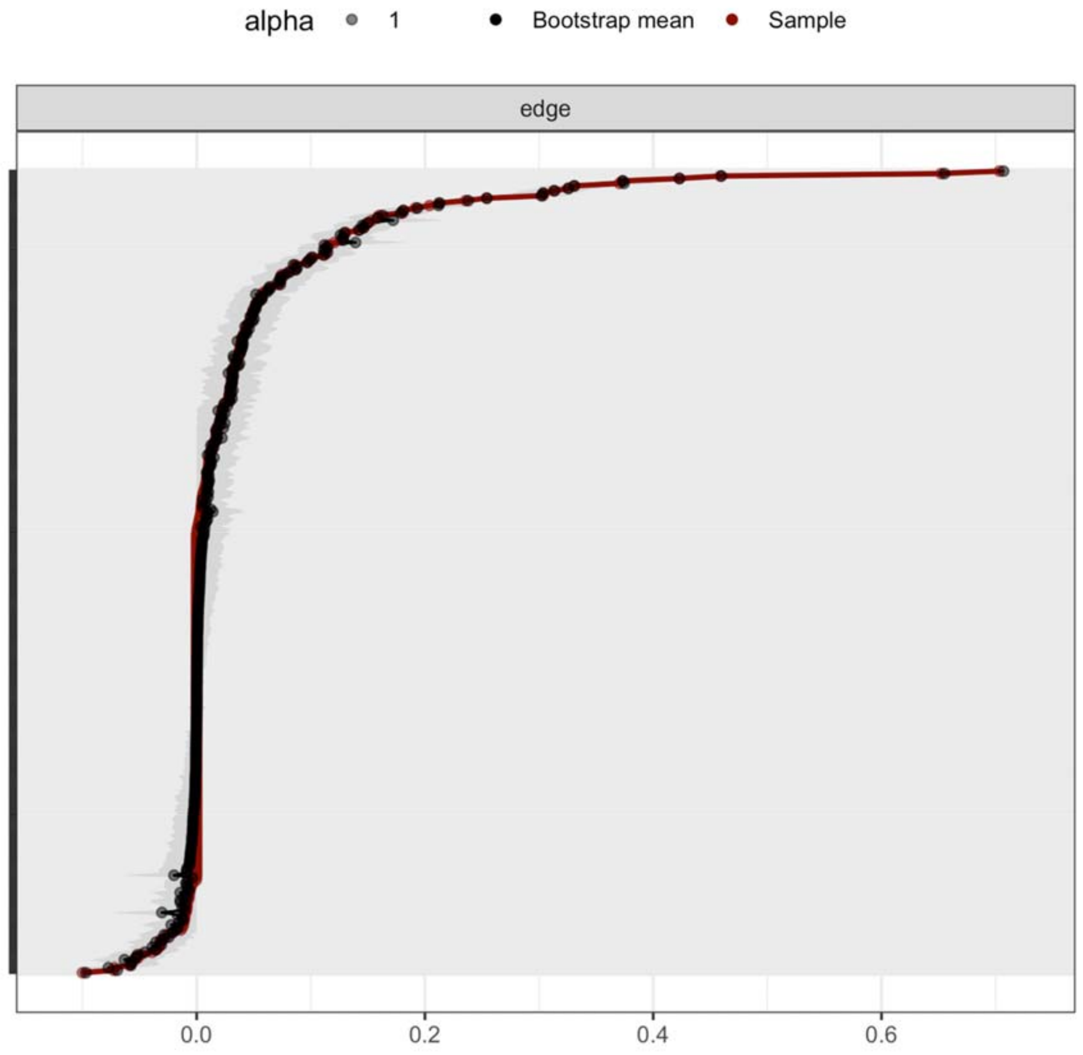


**Figure S5. Edge estimates (red dots) with bootstrapped 95% confidence interval (gray shaded region) for Wave 3. Each dot represents an edge. Edge labels are suppressed to maintain legibility.**

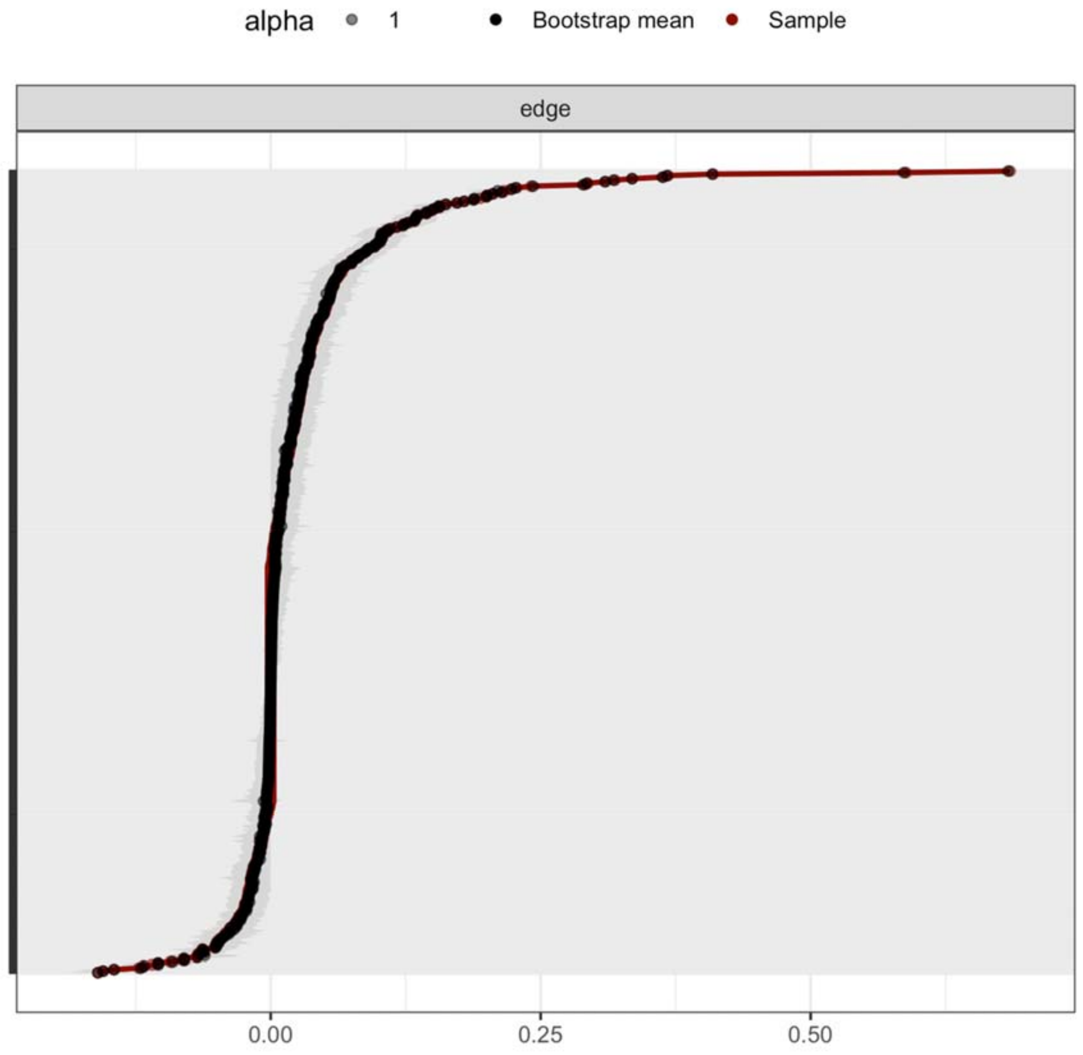


**Figure S6. Edge estimates (red dots) with bootstrapped 95% confidence interval (gray shaded region) for Wave 4. Each dot represents an edge. Edge labels are suppressed to maintain legibility.**

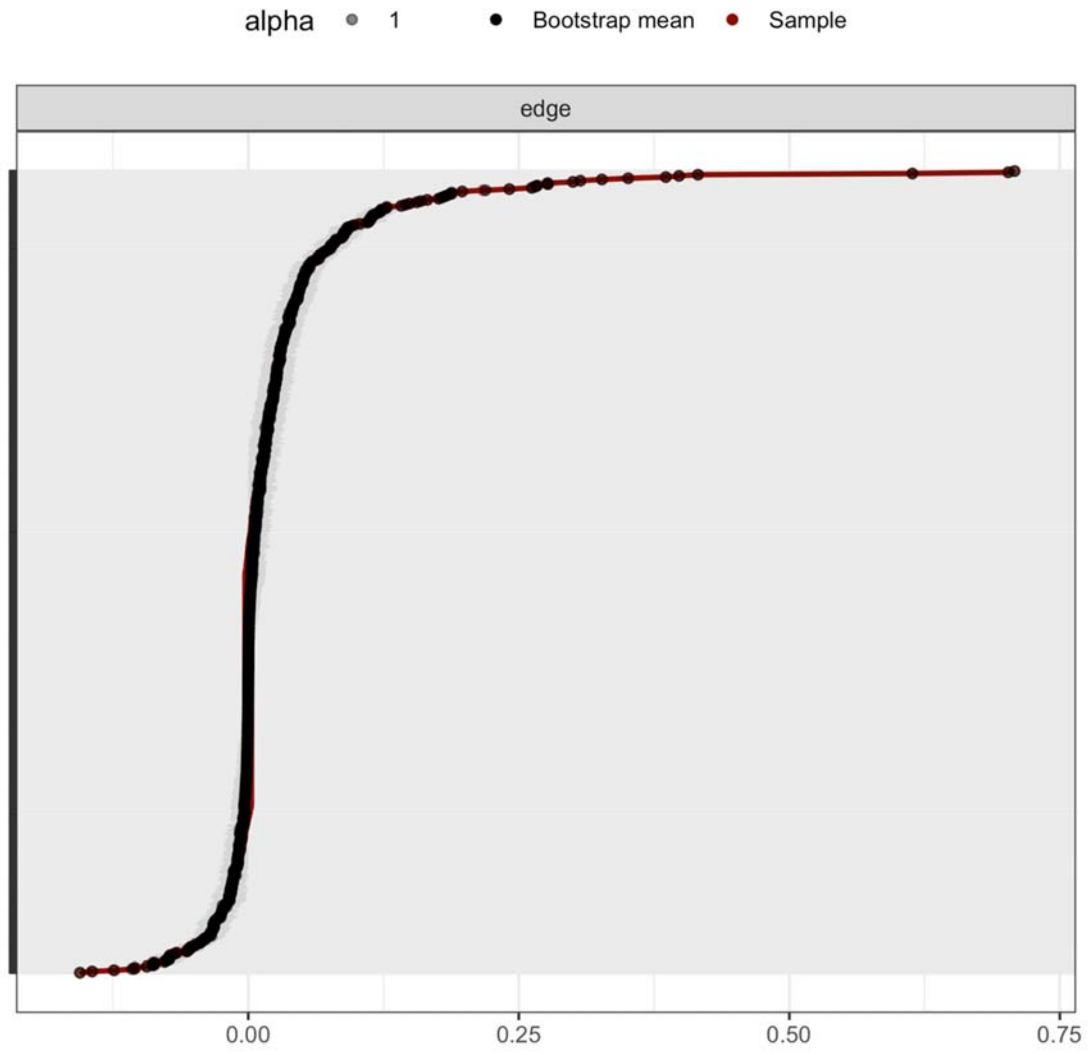


**Figure S7. Edge estimates (red dots) with bootstrapped 95% confidence interval (gray shaded region) for Wave 5. Each dot represents an edge. Edge labels are suppressed to maintain legibility.**

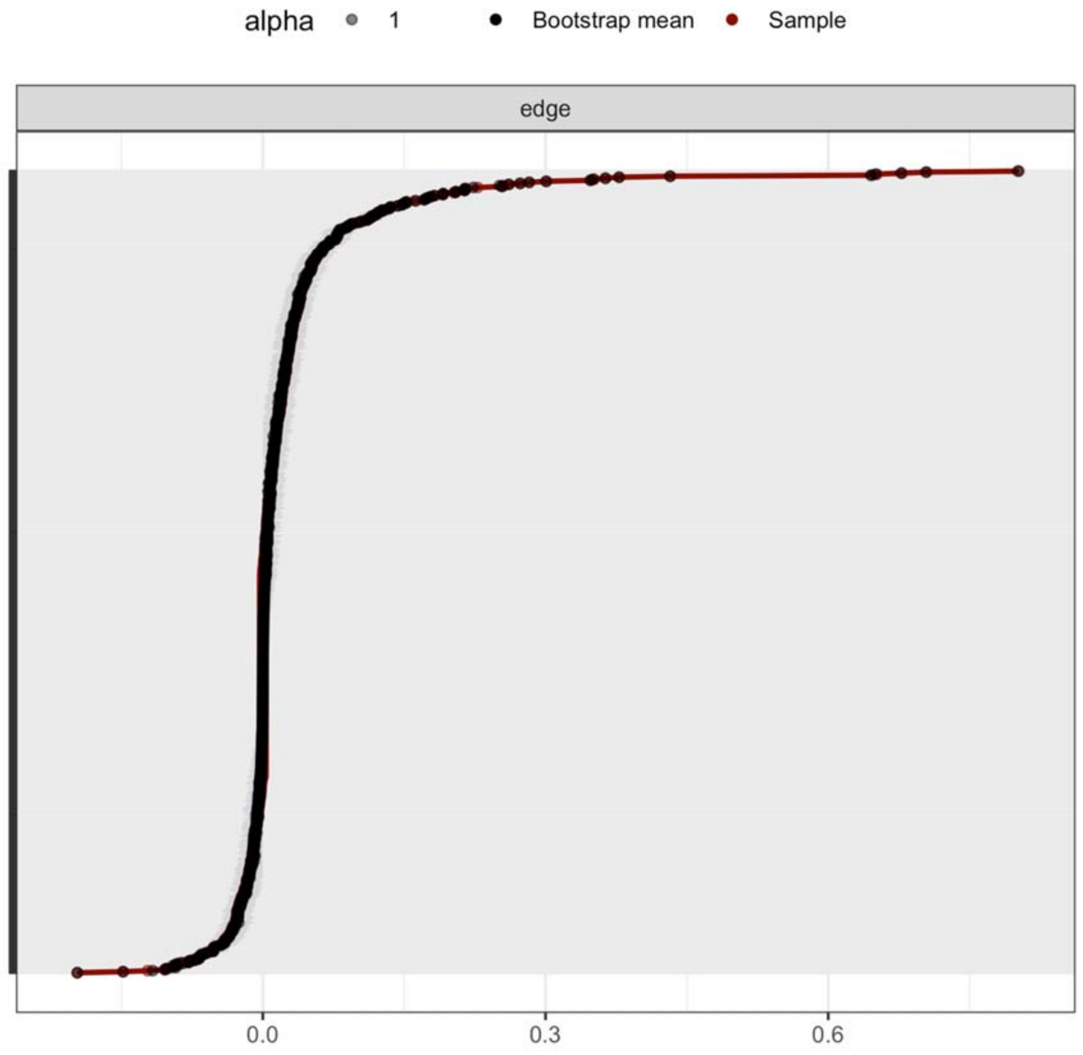


**Figure S8. Edge estimates (red dots) with bootstrapped 95% confidence interval (gray shaded region) for Wave 6. Each dot represents an edge. Edge labels are suppressed to maintain legibility.**

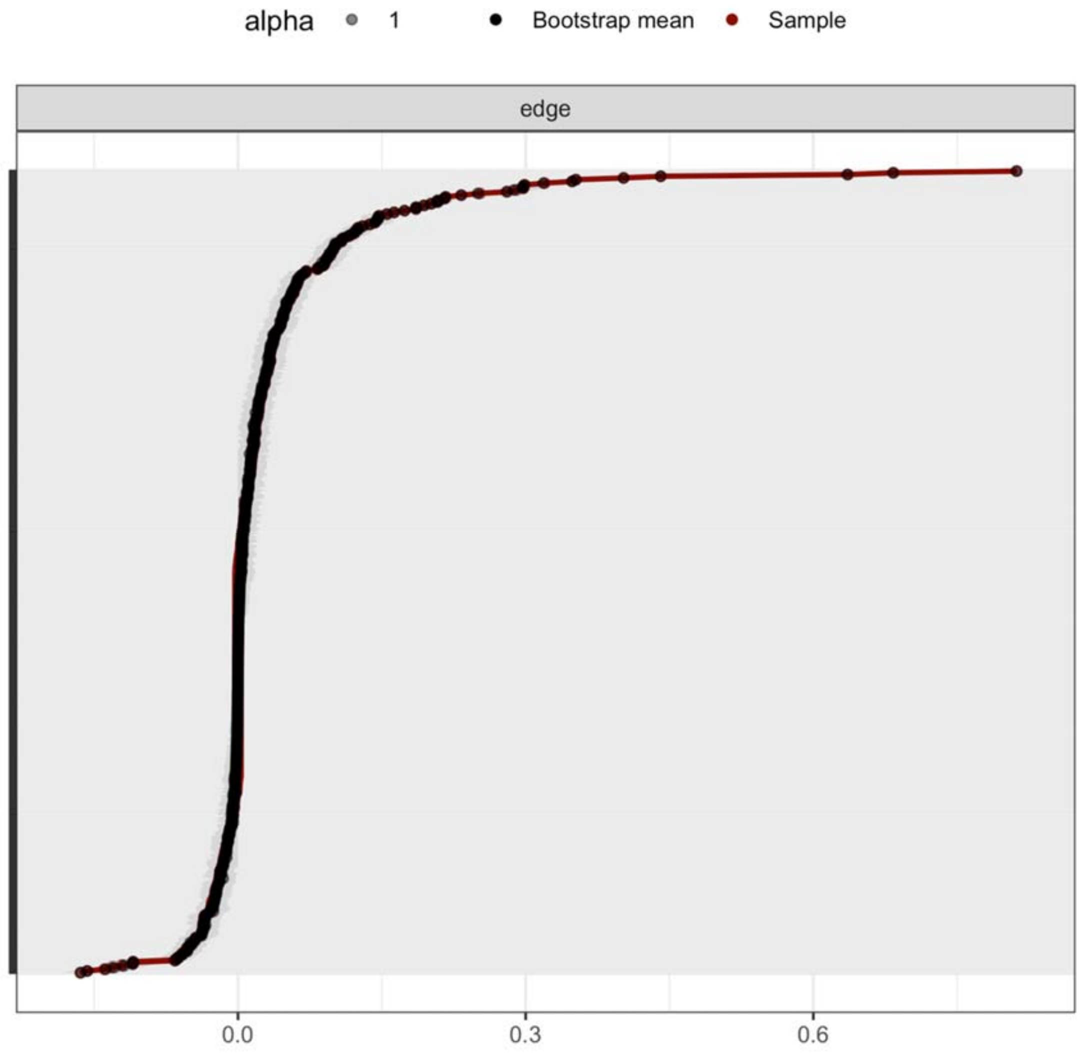


**Figure S9. Edge estimates (red dots) with bootstrapped 95% confidence interval (gray shaded region) for Wave 7. Each dot represents an edge. Edge labels are suppressed to maintain legibility.**

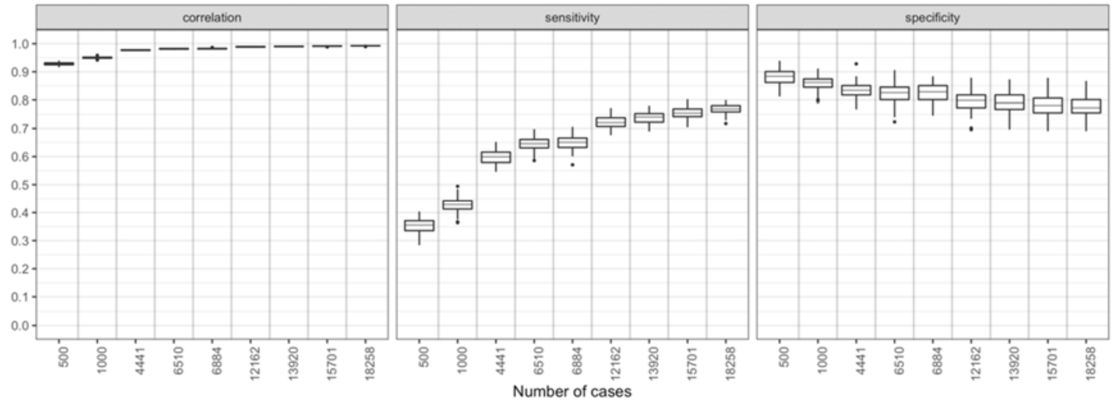


**Figure S10. Network specificity, sensitivity, and edge correlations for simulated networks.**

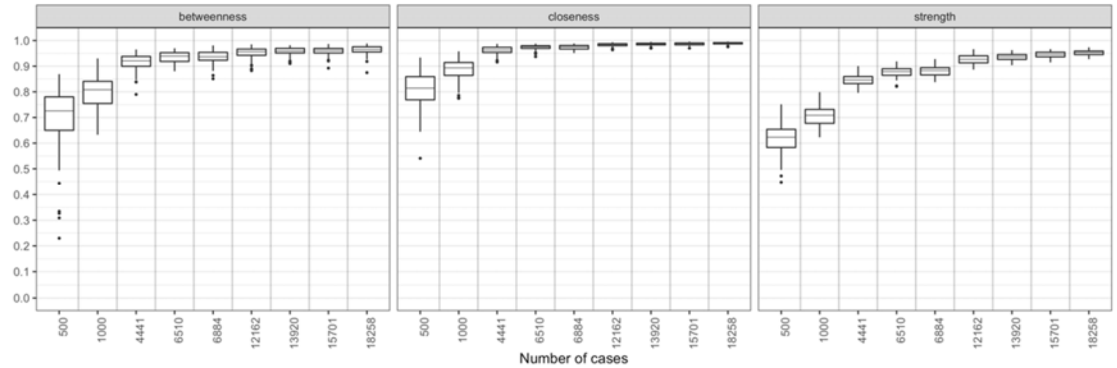


**Figure S11. Correlations between simulated networks and true networks centrality estimates.**

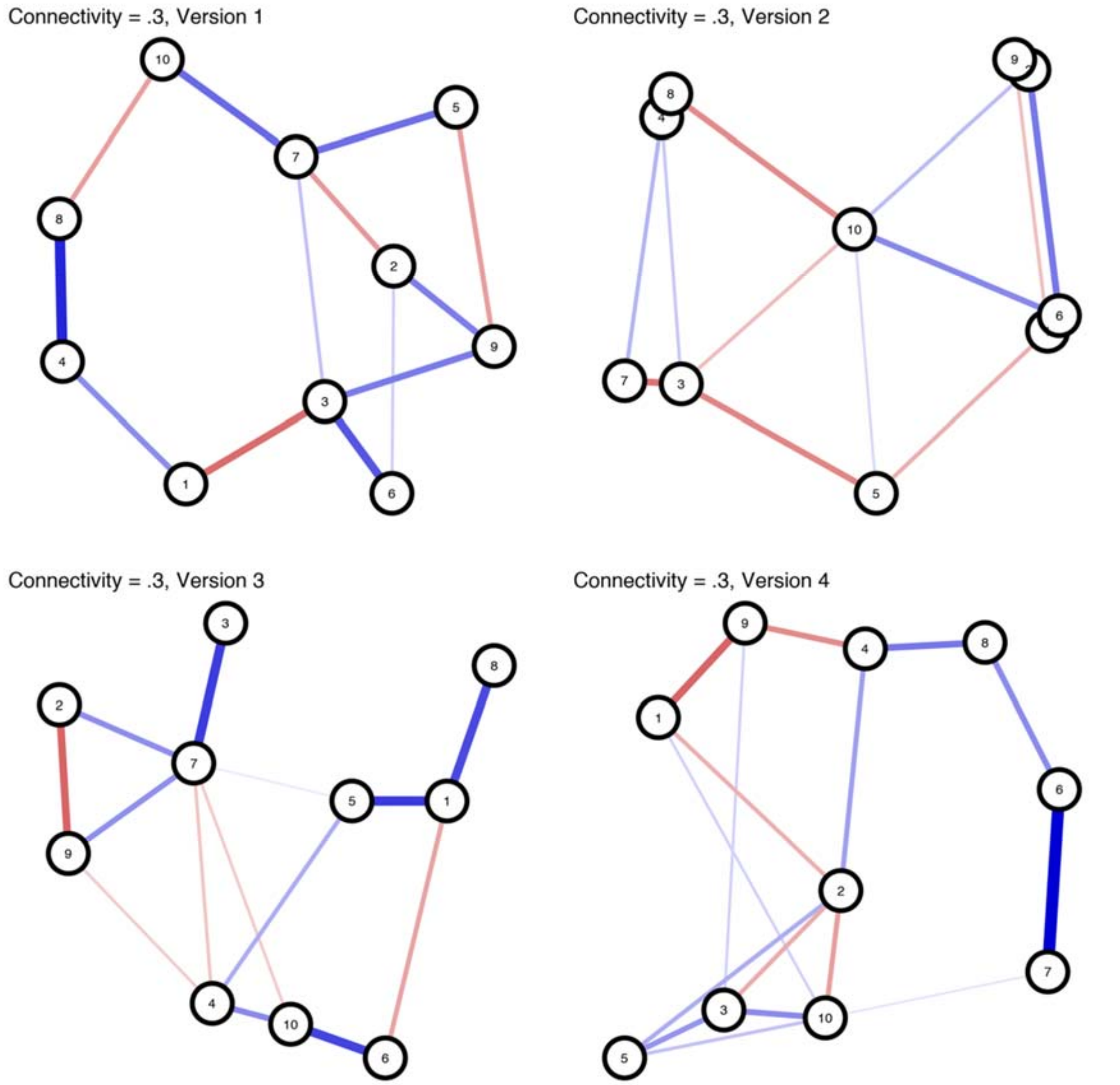


**Figure S12. Lower connectivity "true" networks used for the simulation study. Solid blue edges are positive. Dashed red edges are negative. Thicker edges represent stronger connections between nodes. Placement of the nodes is determined with multidimensional scaling of the absolute value of the adjacency matrix (Jones, Mair, & McNally, 2018).**

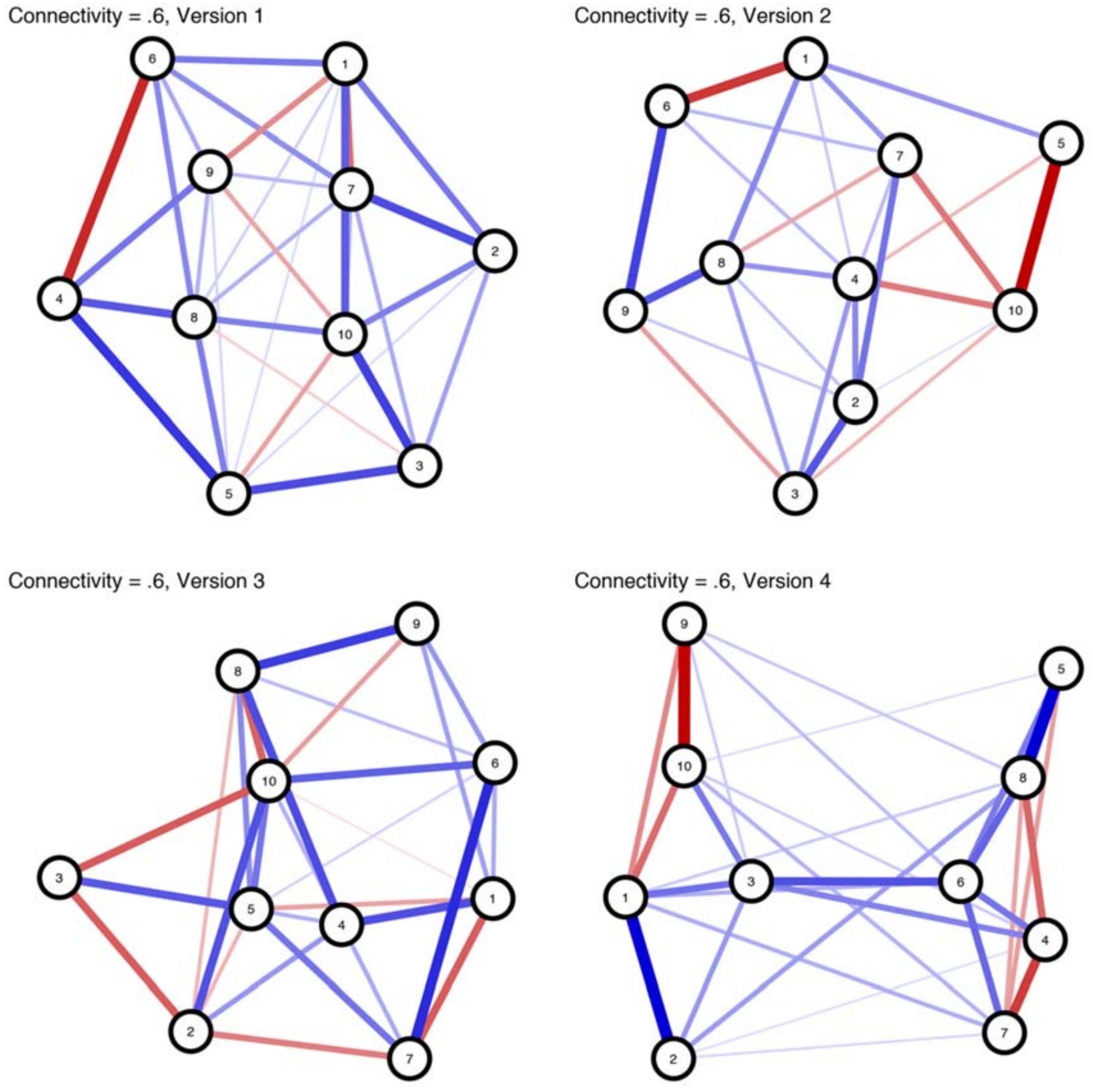


**Figure S13. Higher connectivity "true" networks used for the simulation study. Solid blue edges are positive. Dashed red edges are negative. Thicker edges represent stronger connections between nodes. Placement of the nodes is determined with multidimensional scaling of the absolute value of the adjacency matrix (Jones, Mair, & McNally, 2018).**

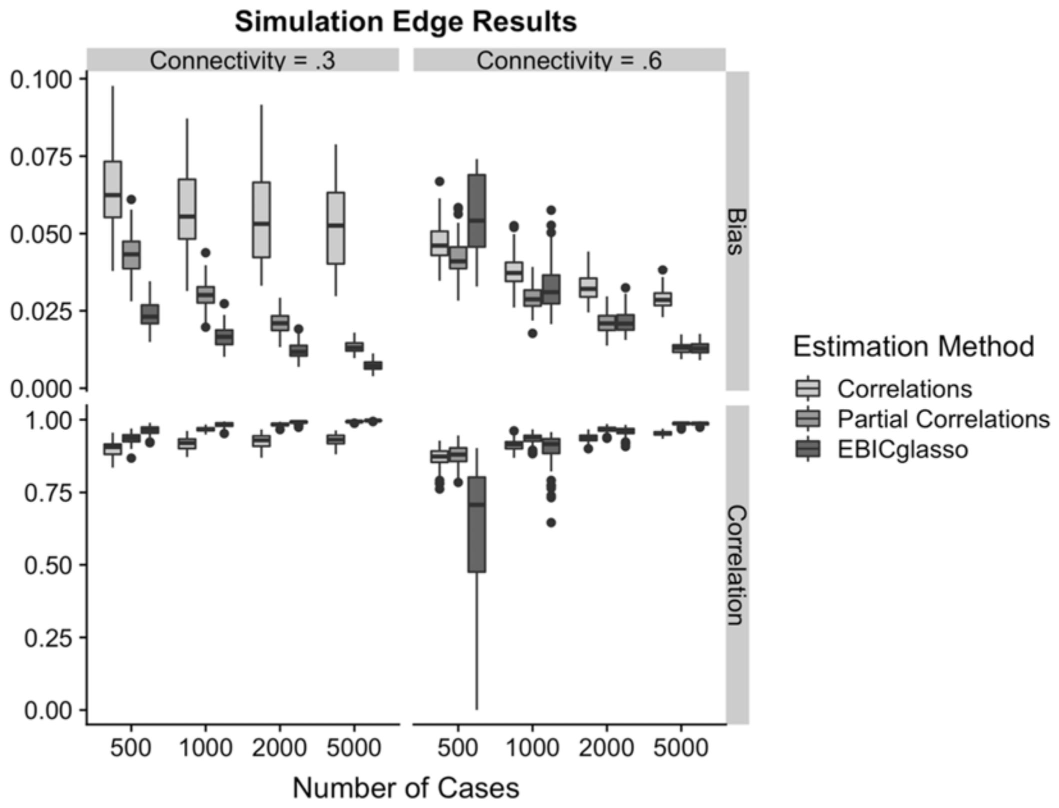


**Figure S14. Edge weight bias and correlations comparing the edges in the true networks to the edges in the simulated networks for each of the three estimation methods.**

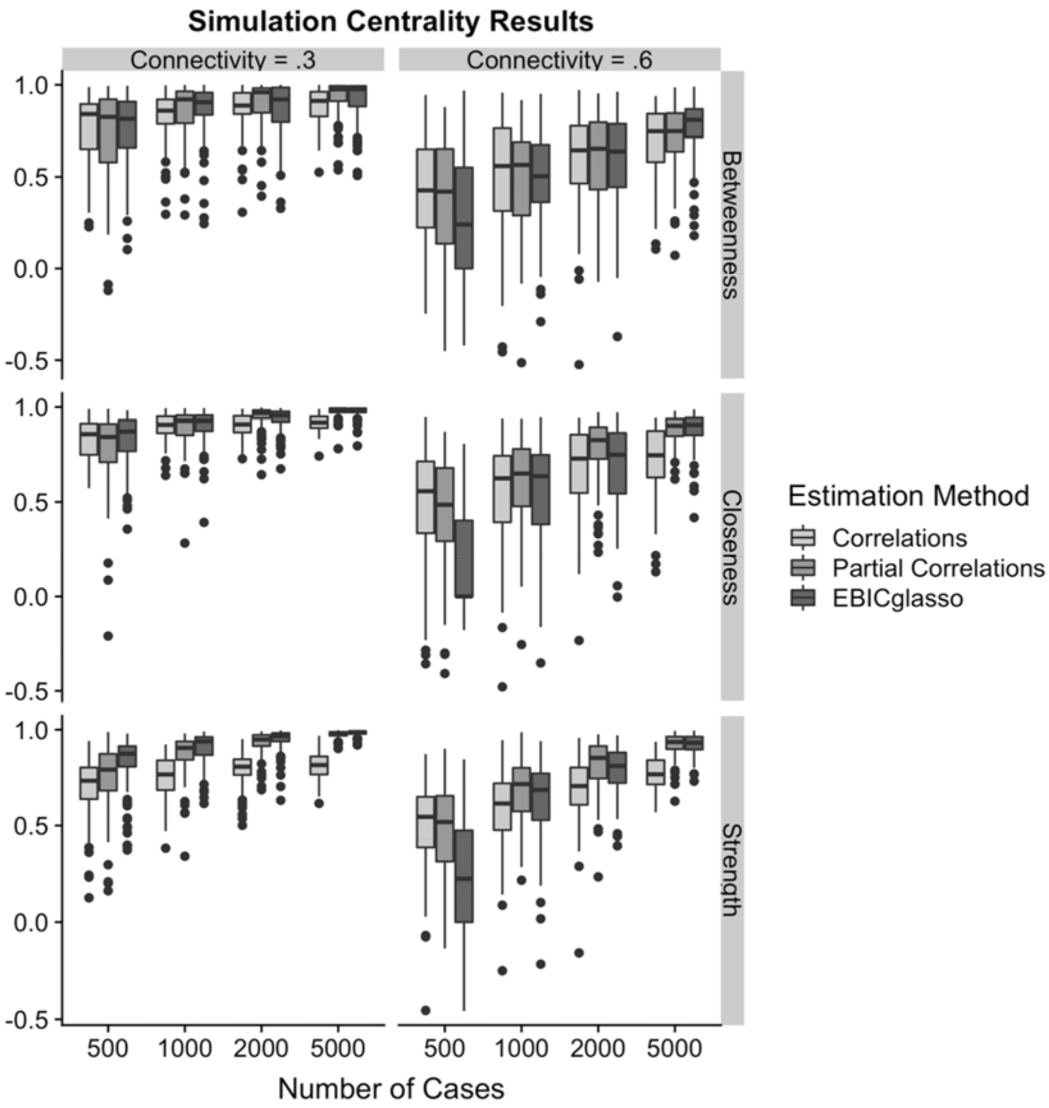


**Figure S15. Correlations comparing centrality estimates in the true networks to the edges in the simulated networks for each of the three estimation methods.**

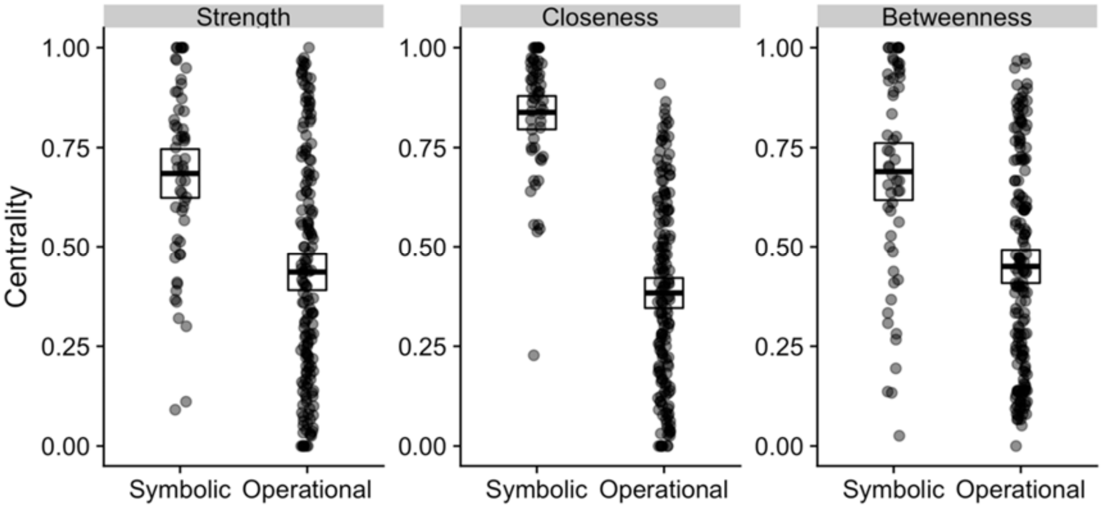


**Figure S16. Symbolic components are higher in strength, closeness, and betweenness centrality than operational components across the belief system networks using standardized measures of centrality. Top and bottom edges of the boxes represent upper and lower bounds of the 95% confidence interval of the mean. The centerline of the box represents the mean.**

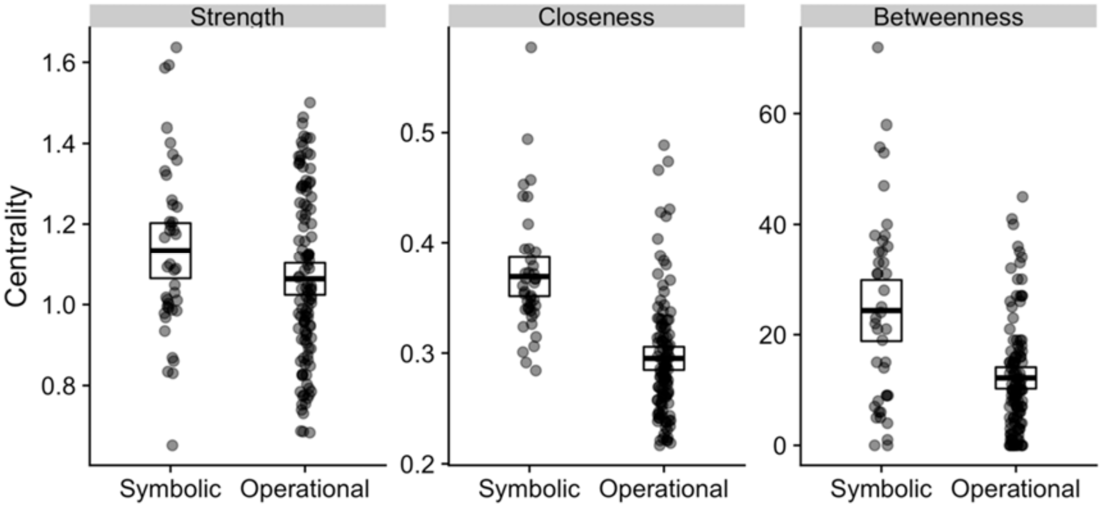


**Figure S17. Symbolic components are higher in closeness and betweenness centrality than operational components across the belief system networks that only include nodes found in at least 6 of the waves. Strength shows similar findings, but they are not as clear. Top and bottom edges of the boxes represent upper and lower bounds of the 95% confidence interval of the mean. The centerline of the box represents the mean.**

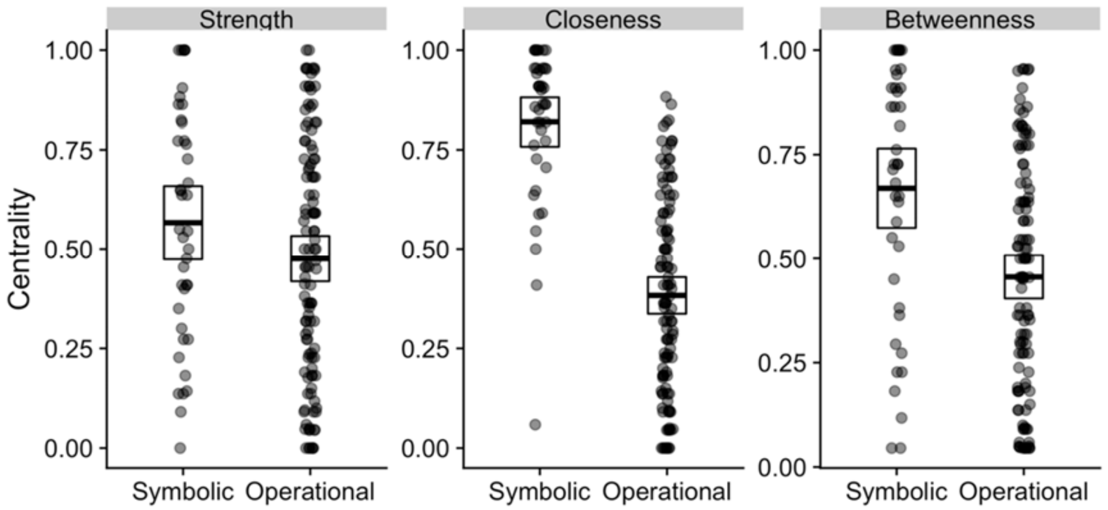


**Figure S18. Symbolic components are higher in closeness and betweenness centrality than operational components across the belief system networks that only include nodes found in at least 6 of the waves and using standardized measures of centrality. Strength shows similar findings, but they are not as clear. Top and bottom edges of the boxes represent upper and lower bounds of the 95% confidence interval of the mean. The centerline of the box represents the mean.**

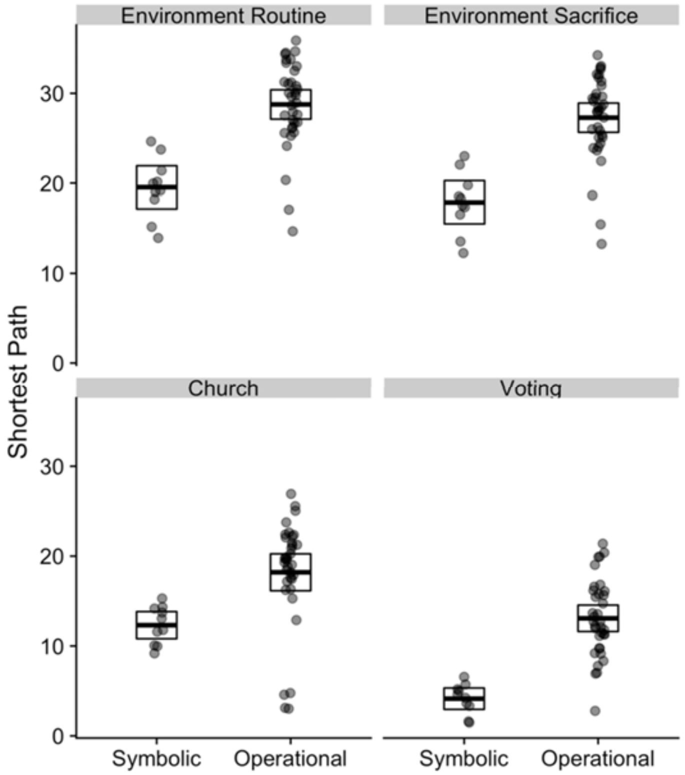


**Figure S19. Symbolic components are more closely connected (have shorter paths) with behaviors than operational components in networks that only include nodes found in at least 6 of the waves. Top and bottom edges of the boxes represent upper and lower bounds of the 95% confidence interval of the mean. The centerline of the box represents the mean.**

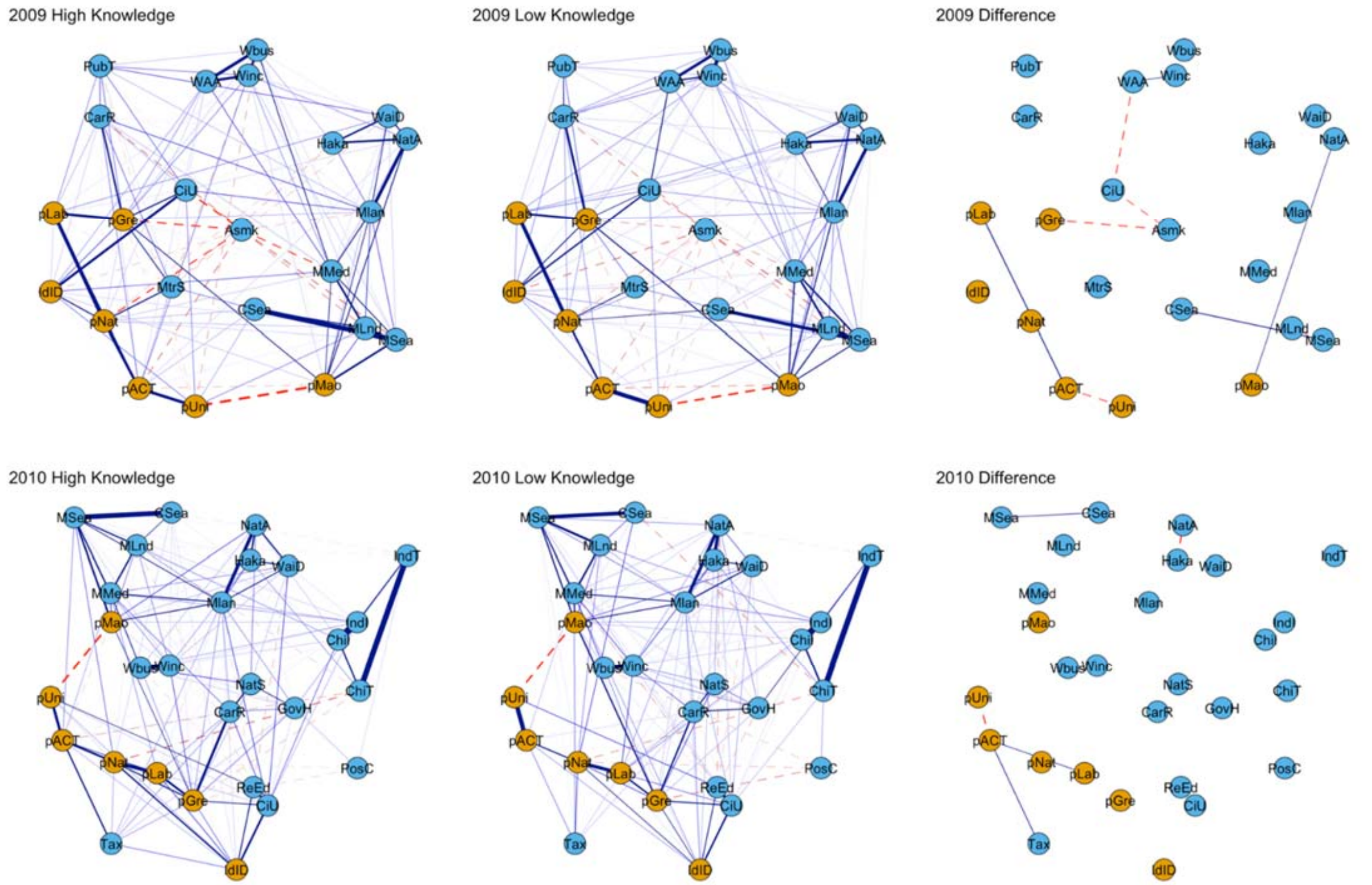


**Figure S20. Belief system networks with symbolic (gold nodes) and operational (blue nodes) components for Waves 1 & 2 for people with high and low levels of political knowledge. Solid blue edges are positive. Dashed red edges are negative. Thicker edges represent stronger connections between nodes. Placement of the nodes is the same as figures from the complete sample. Difference networks plot differences between high and low political knowledge nodes that exceed |.10|.**

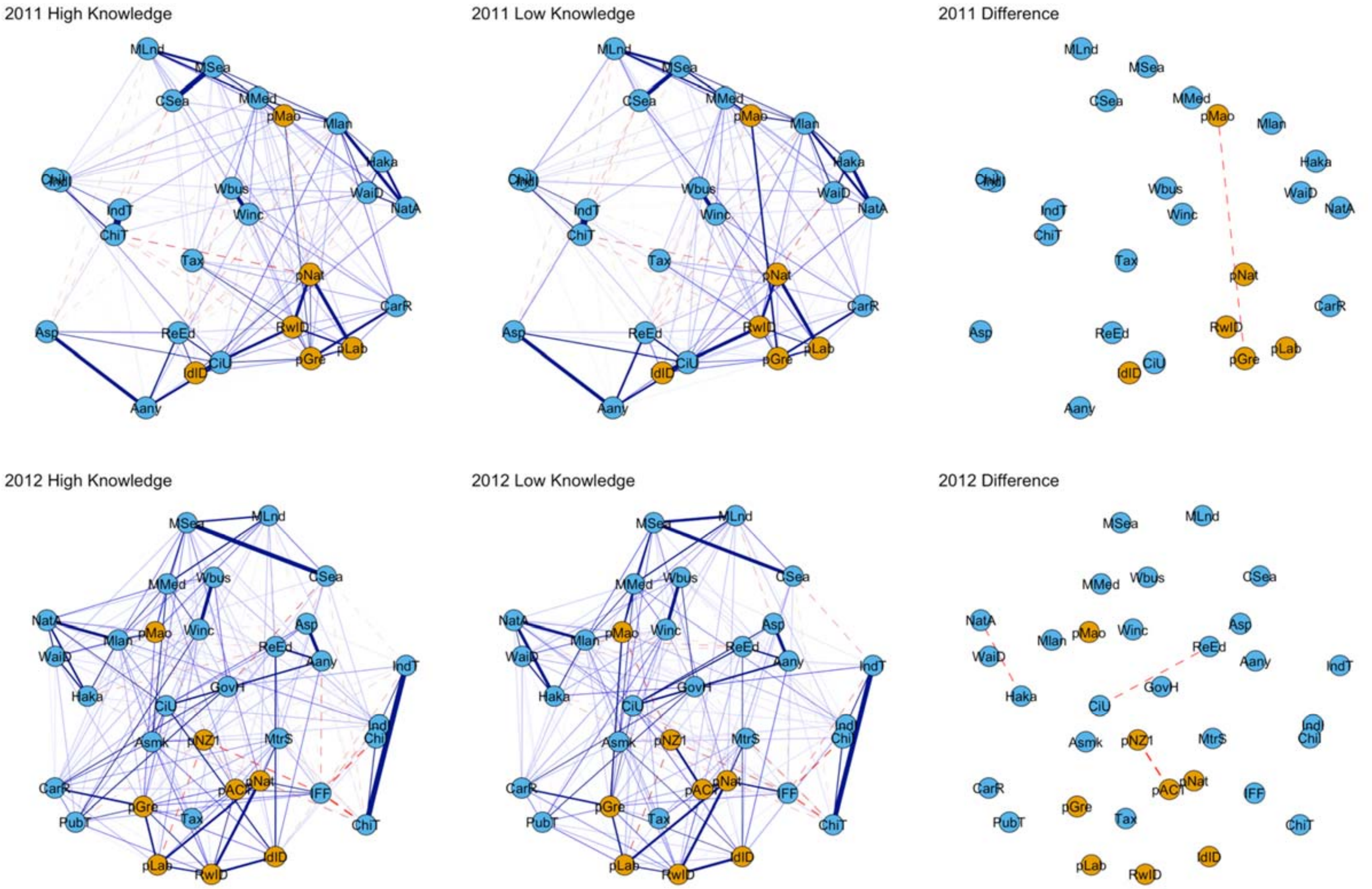


**Figure S21. Belief system networks with symbolic (gold nodes) and operational (blue nodes) components for Waves 3 & 4 for people with high and low levels of political knowledge. Solid blue edges are positive. Dashed red edges are negative. Thicker edges represent stronger connections between nodes. Placement of the nodes is the same as figures from the complete sample. Difference networks plot differences between high and low political knowledge nodes that exceed |.10|.**

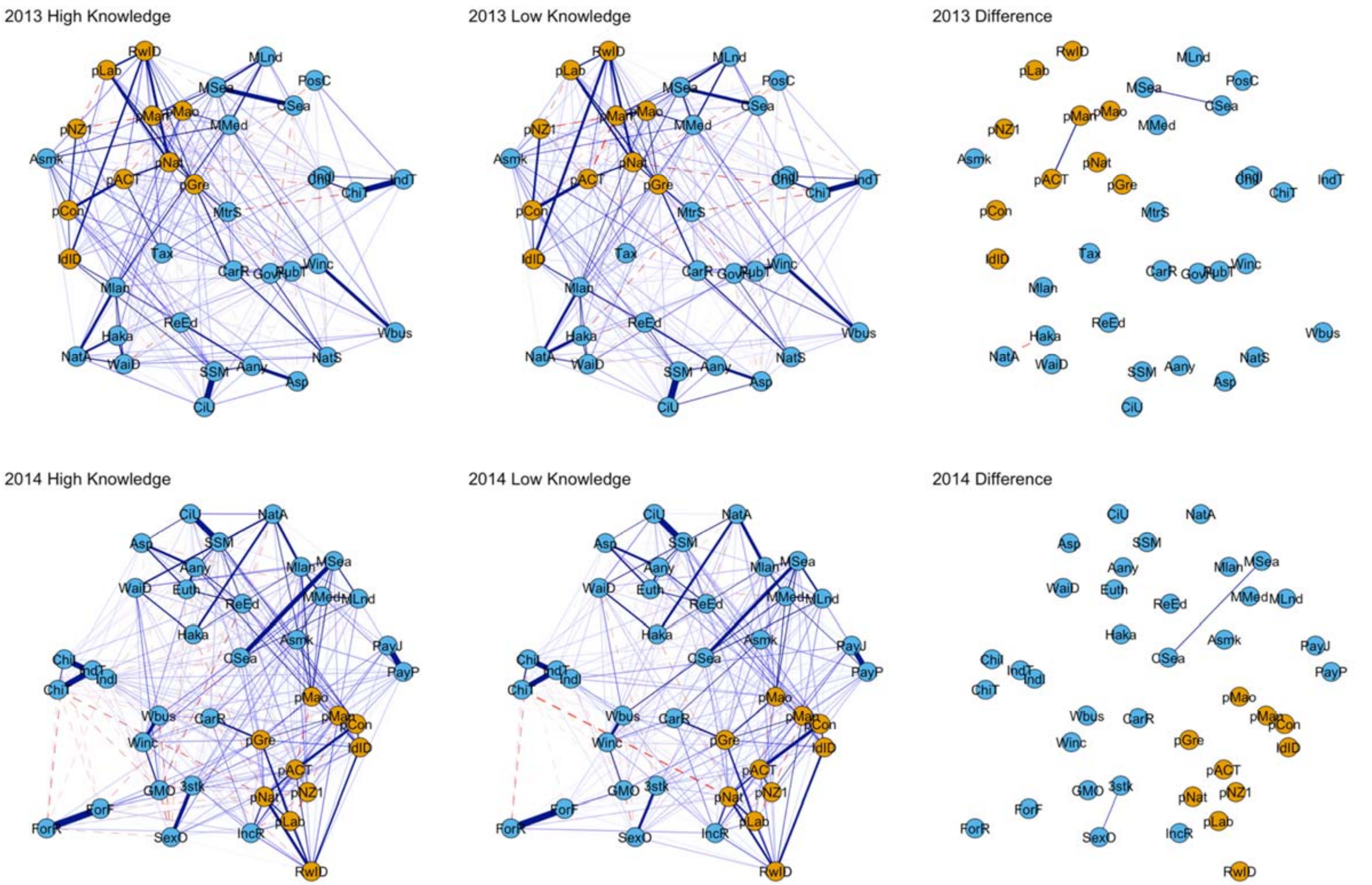


**Figure S22. Belief system networks with symbolic (gold nodes) and operational (blue nodes) components for Waves 5 & 6 for people with high and low levels of political knowledge. Solid blue edges are positive. Dashed red edges are negative. Thicker edges represent stronger connections between nodes. Placement of the nodes is the same as figures from the complete sample. Difference networks plot differences between high and low political knowledge nodes that exceed |.10|.**

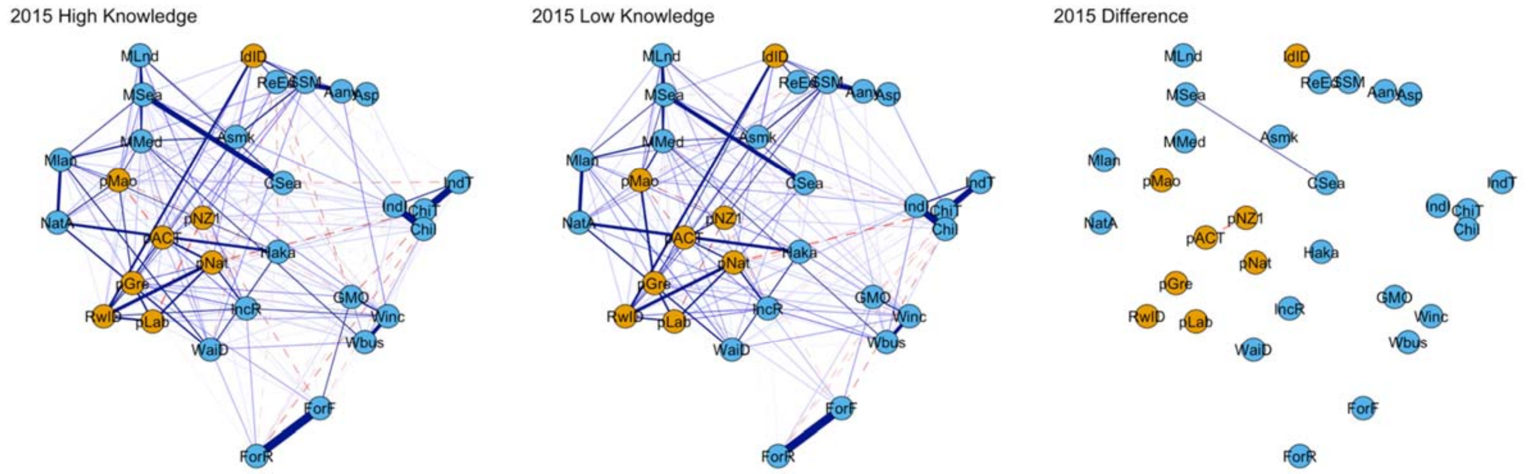


**Figure S23. Belief system networks with symbolic (gold nodes) and operational (blue nodes) components for Wave 7 for people with high and low levels of political knowledge. Solid blue edges are positive. Dashed red edges are negative. Thicker edges represent stronger connections between nodes. Placement of the nodes is the same as figures from the complete sample. Difference network plots differences between high and low political knowledge nodes that exceed |.10|**

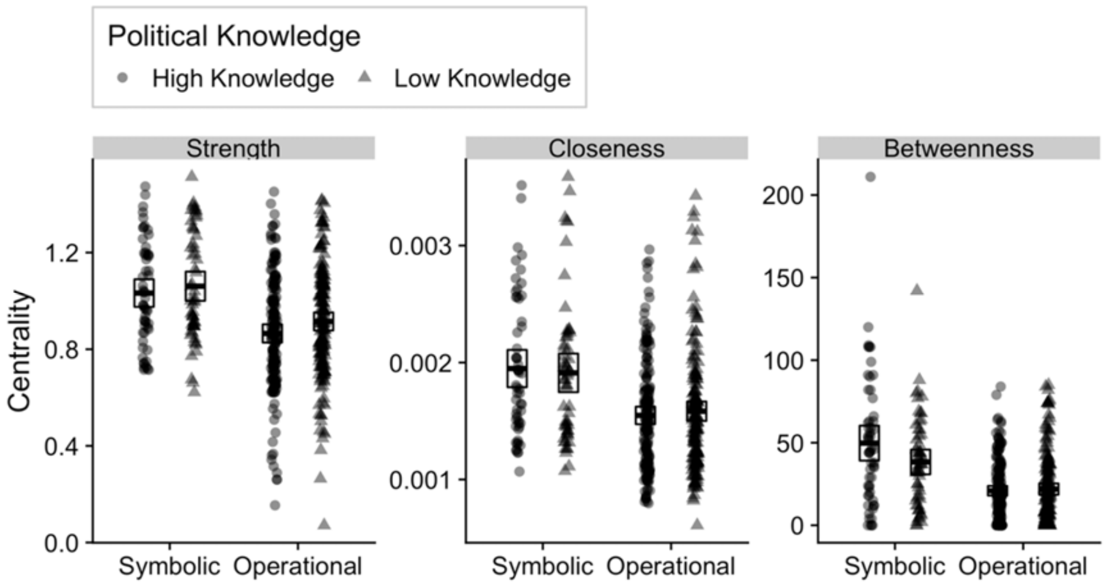


**Figure S24. Symbolic components are higher in centrality than operational components across the belief system networks. The pattern is the same for people high and low in political knowledge. Top and bottom edges of the boxes represent upper and lower bounds of the 95% confidence interval of the mean. The centerline of the box represents the mean.**

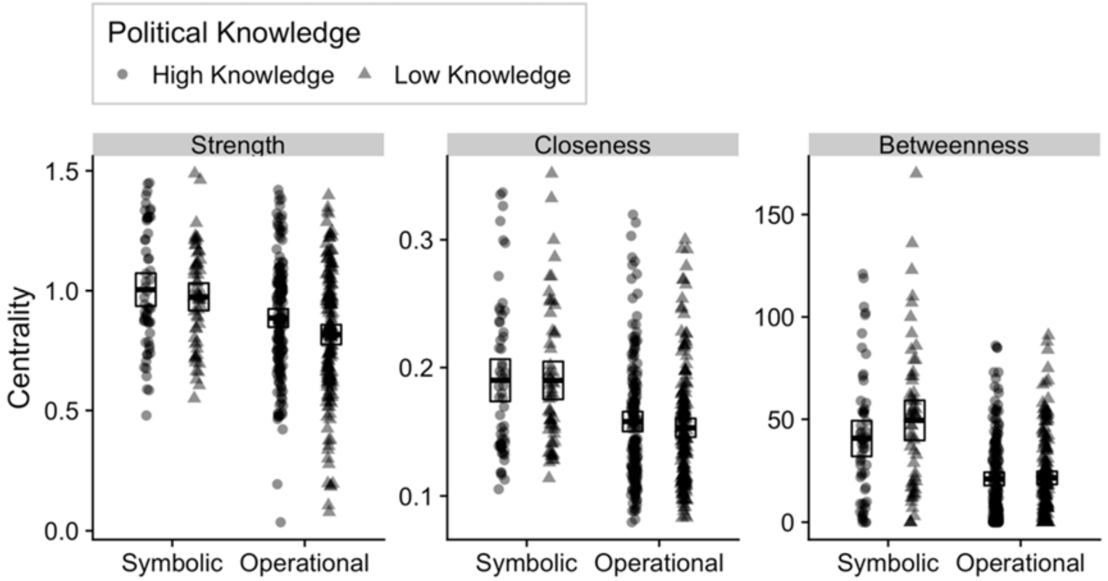


**Figure S25. Symbolic components are higher in centrality than operational components across the belief system networks. The pattern is the same for people high and low in political knowledge when using people from the top and bottom quartiles of political knowledge. Top and bottom edges of the boxes represent upper and lower bounds of the 95% confidence interval of the mean. The centerline of the box represents the mean.**

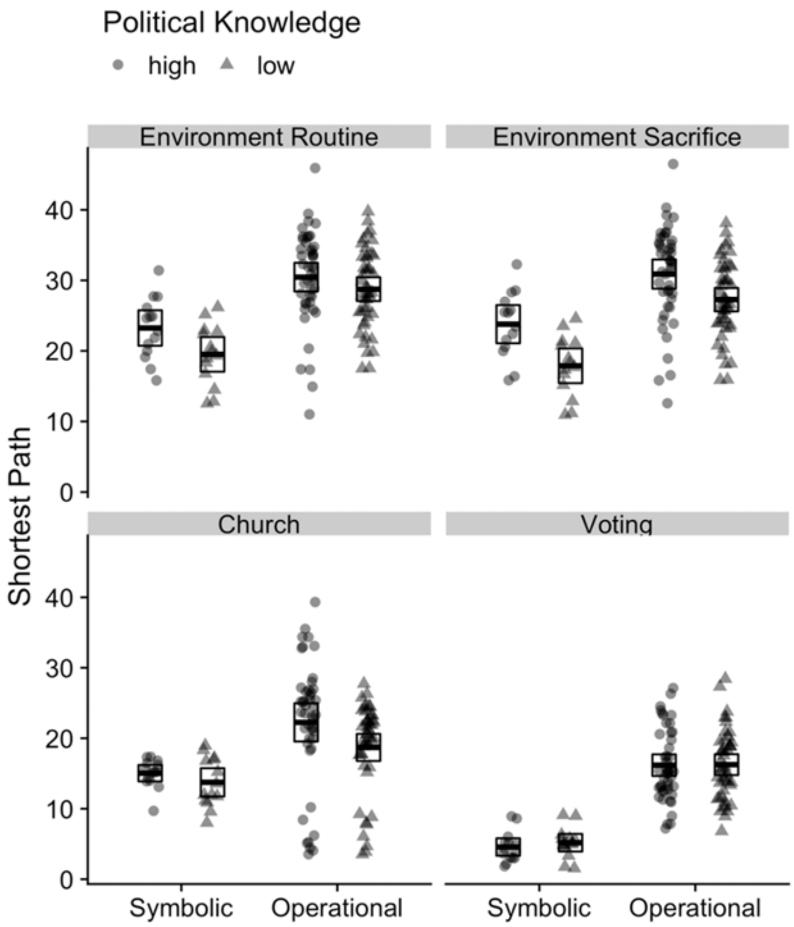


**Figure S26. Symbolic components are more closely connected (have shorter paths) with behaviors than operational components for people with both high and low levels of political knowledge. Top and bottom edges of the boxes represent upper and lower bounds of the 95% confidence interval of the mean. The centerline of the box represents the mean.**

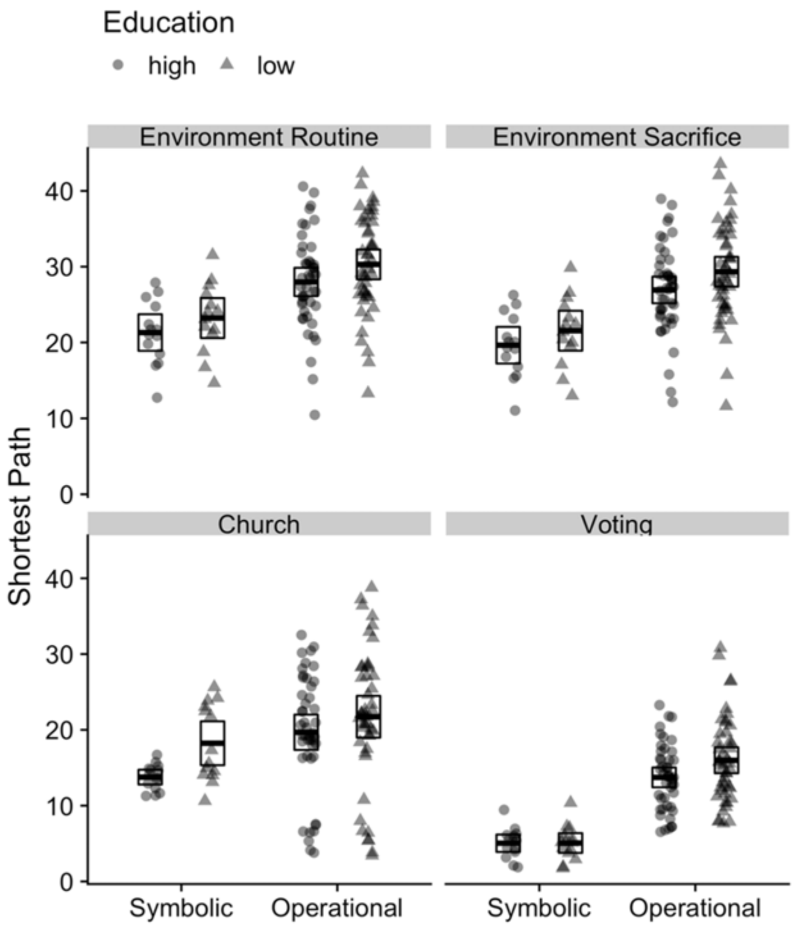


**Figure S27. Symbolic components are more closely connected (have shorter paths) with behaviors than operational components for people with both high and low levels of education. Top and bottom edges of the boxes represent upper and lower bounds of the 95% confidence interval of the mean. The centerline of the box represents the mean.**

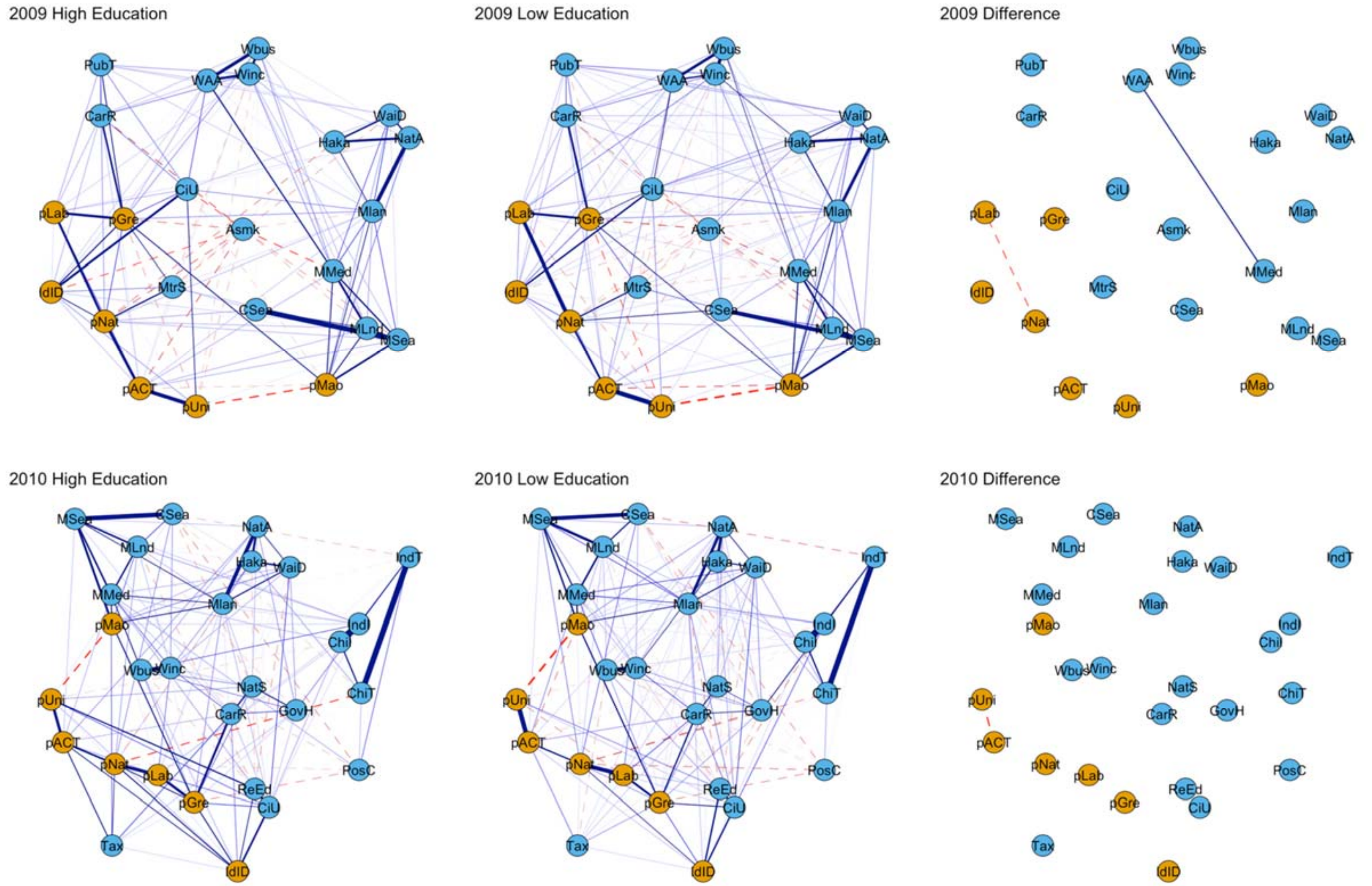


**Figure S28. Belief system networks with symbolic (gold nodes) and operational (blue nodes) components for Waves 1 & 2 for people with high and low levels of education. Solid blue edges are positive. Dashed red edges are negative. Thicker edges represent stronger connections between nodes. Placement of the nodes is the same as figures from the complete sample. Difference networks plot differences between high and low education nodes that exceed |.10|.**

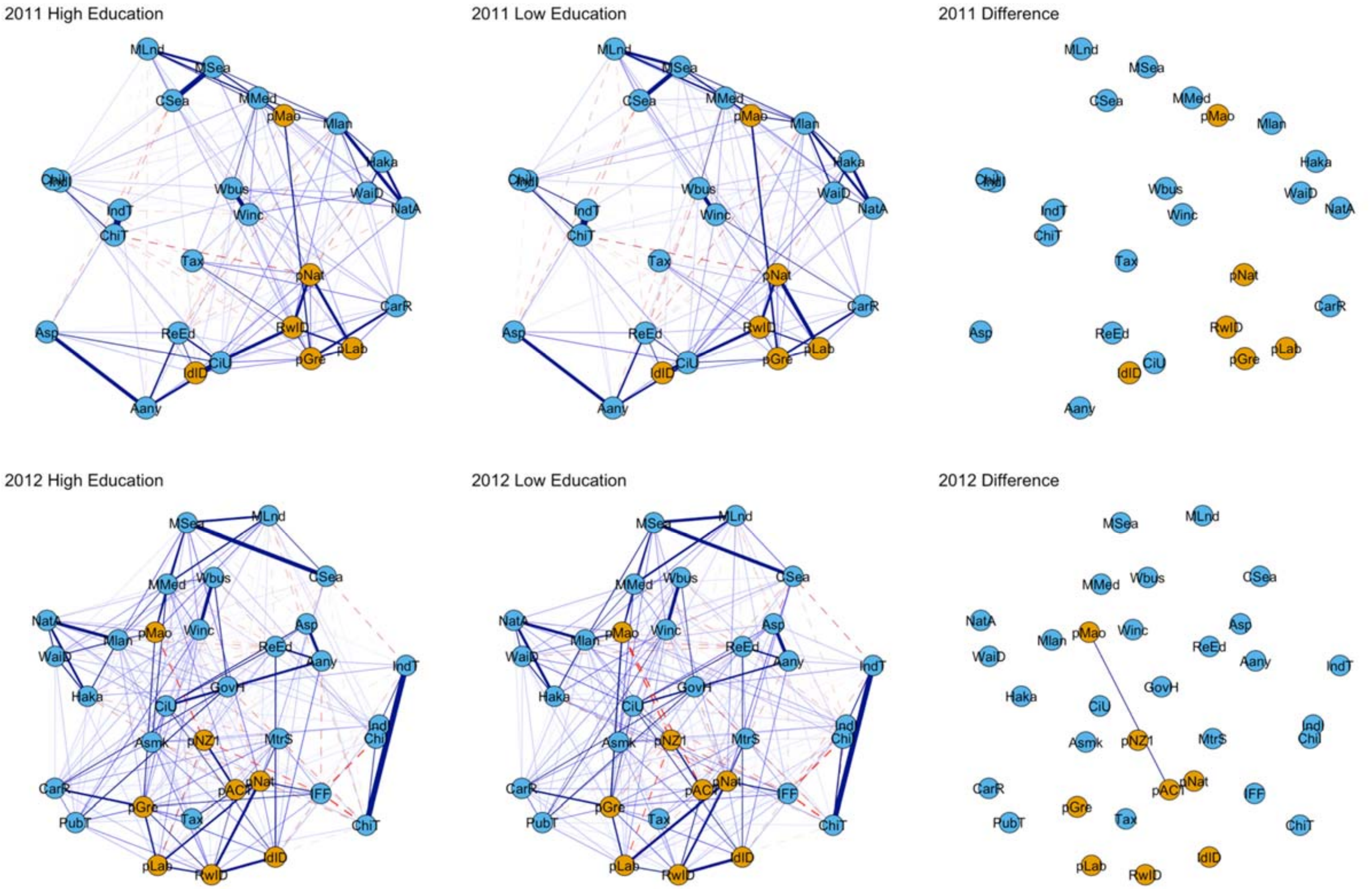


Figure S29. Belief system networks with symbolic (gold nodes) and operational (blue nodes) components for Waves 3 & 4 for people with high and low levels of education. Solid blue edges are positive. Dashed red edges are negative. Thicker edges represent stronger connections between nodes. Placement of the nodes is the same as figures from the complete sample. Difference networks plot differences between high and low education nodes that exceed |.10|.

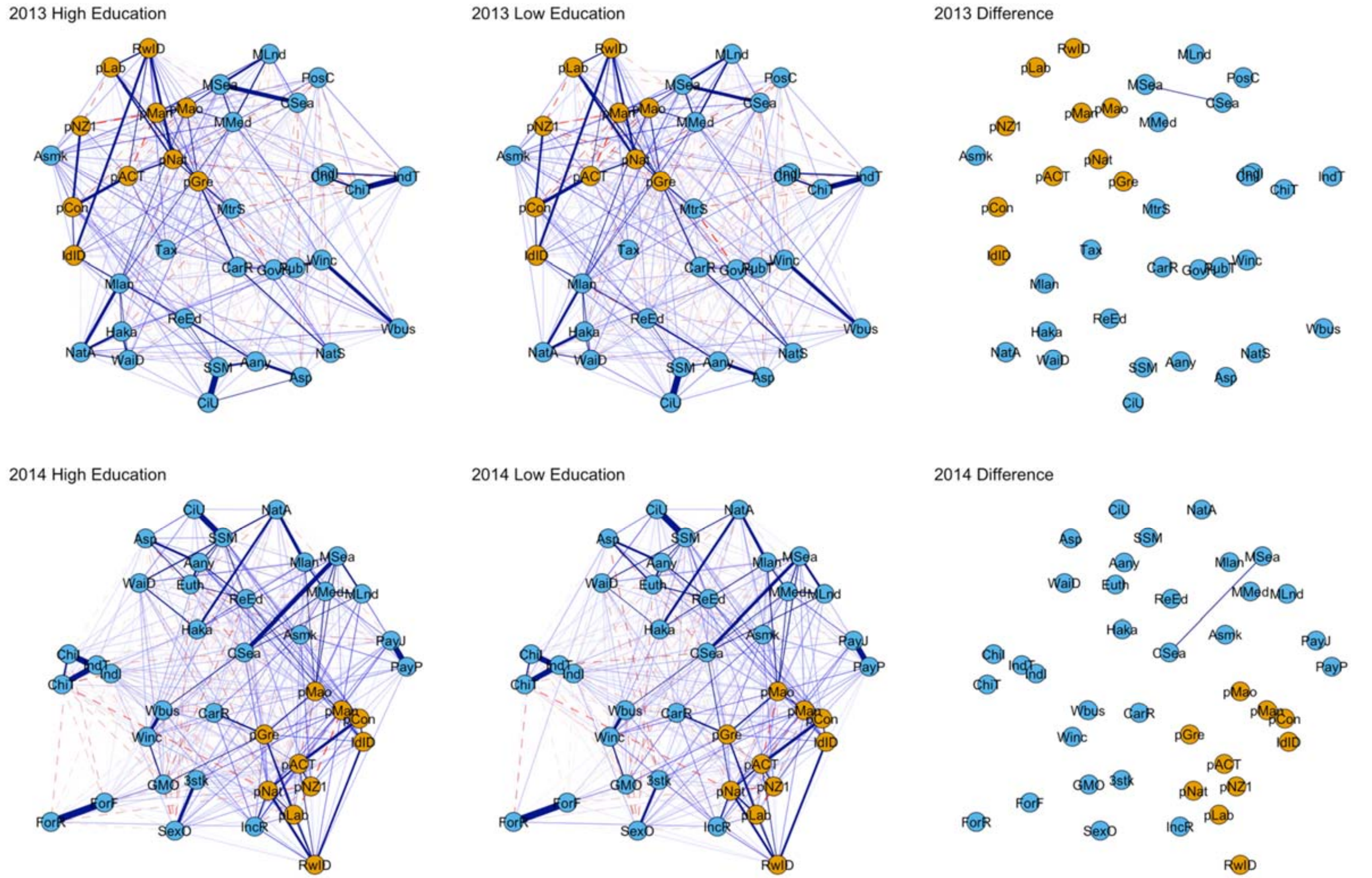


**Figure S30. Belief system networks with symbolic (gold nodes) and operational (blue nodes) components for Waves 5 & 6 for people with high and low levels of education. Solid blue edges are positive. Dashed red edges are negative. Thicker edges represent stronger connections between nodes. Placement of the nodes is the same as figures from the complete sample. Difference networks plot differences between high and low education nodes that exceed |.10|.**

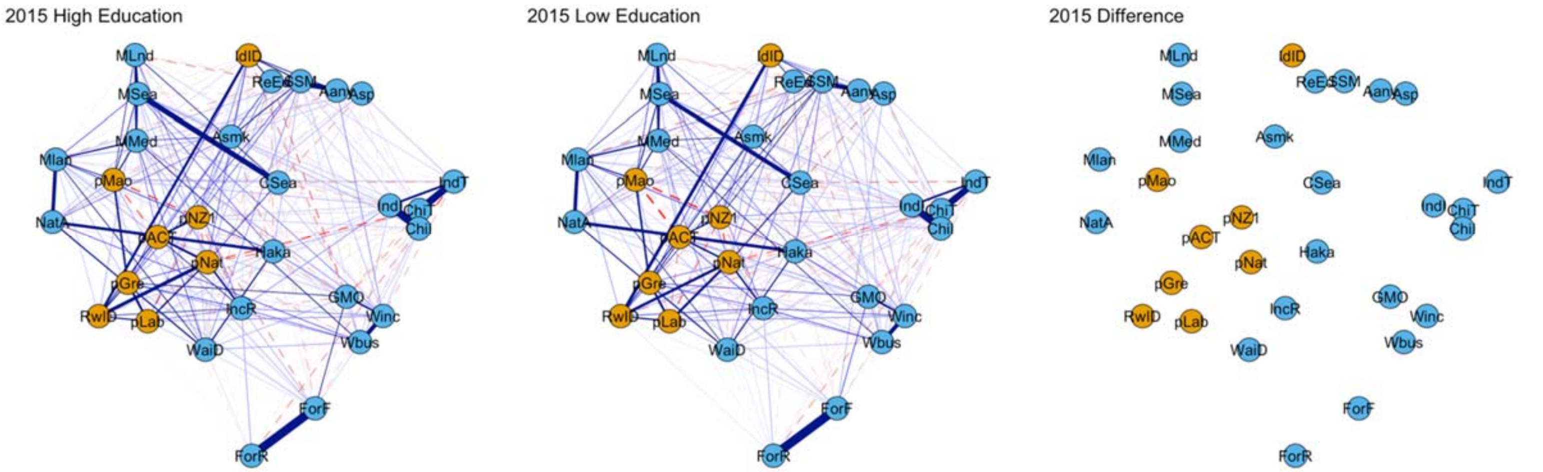


**Figure S31. Belief system networks with symbolic (gold nodes) and operational (blue nodes) components for Wave 7 for people with high and low levels of education. Solid blue edges are positive. Dashed red edges are negative. Thicker edges represent stronger connections between nodes. Placement of the nodes is the same as figures from the complete sample. Difference network plots differences between high and low education nodes that exceed |.10|.**

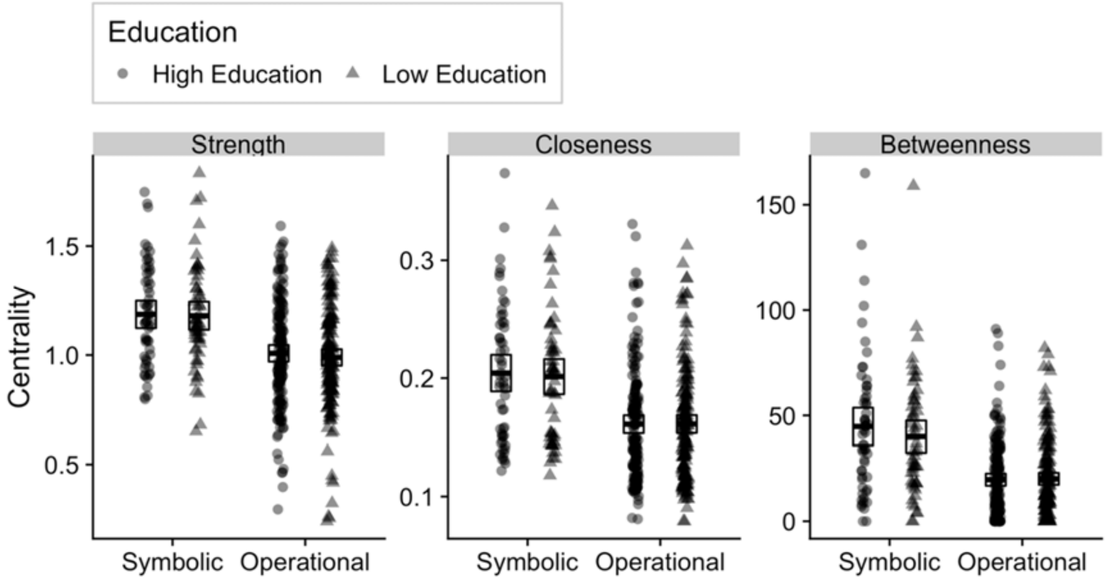


**Figure S32. Symbolic components are higher in centrality than operational components across the belief system networks. The pattern is the same for people high and low in political knowledge. Top and bottom edges of the boxes represent upper and lower bounds of the 95% confidence interval of the mean. The centerline of the box represents the mean.**

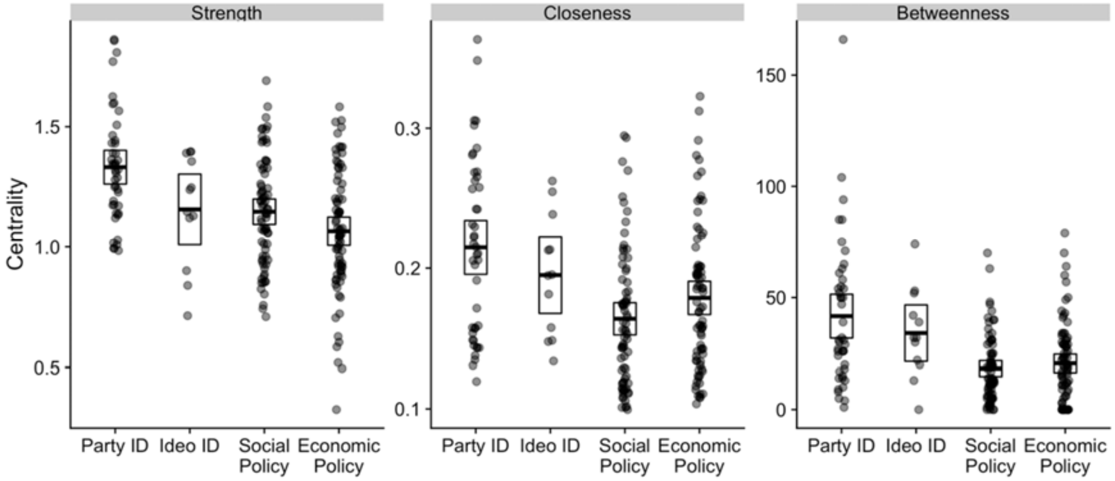


**Figure S33. Symbolic components are higher in centrality than operational components across the belief system networks. This is most often true of party attachments. Top and bottom edges of the boxes represent upper and lower bounds of the 95% confidence interval of the mean. The centerline of the box represents the mean.**

## Tables

**Table S1. Percentage of missing data per item per wave.**

| Label | Wave 1 | Wave 2 | Wave 3 | Wave 4 | Wave 5 | Wave 6 | Wave 7 |
|---|---|---|---|---|---|---|---|
| IdID | 6.5 | 6.7 | 5.9 | 8.3 | 8.9 | 7.2 | 6.5 |
| pNat | 5.3 | 4.2 | 4.4 | 16.6 | 4.8 | 3.1 | 1.5 |
| pLab | 5.8 | 4.7 | 4.6 | 16.7 | 5.2 | 3.2 | 1.9 |
| pGre | 6.6 | 5.2 | 5.0 | 17.1 | 5.3 | 3.2 | 2.0 |
| pMao | 6.7 | 5.6 | 5.4 | 17.4 | 5.7 | 3.4 | 2.1 |
| pACT | 7.5 | 5.9 | | 17.4 | 5.9 | 3.5 | 2.3 |
| pUni | 7.8 | 6.3 | | | | | |
| CiU | 3.8 | 3.0 | 1.8 | 9.7 | 1.7 | 1.6 | |
| Asmk | 1.5 | | | 9.0 | 1.1 | 1.1 | 0.6 |
| MSea | 1.8 | 1.3 | 1.2 | 1.2 | 1.2 | 1.0 | 0.7 |
| MMed | 1.2 | 1.3 | 1.0 | 1.2 | 1.1 | 1.0 | 0.8 |
| MLnd | 1.8 | 1.7 | 1.3 | 1.5 | 1.4 | 1.3 | 0.8 |
| CSea | 1.3 | 1.5 | 0.9 | 1.4 | 1.2 | 1.3 | 0.9 |
| Haka | 1.4 | 1.6 | 0.9 | 1.2 | 1.1 | 0.8 | 0.4 |
| WaiD | 1.7 | 1.6 | 1.1 | 1.5 | 1.3 | 1.1 | 0.8 |
| Mlan | 1.4 | 1.4 | 1.0 | 1.3 | 1.1 | 0.9 | 0.8 |
| NatA | 0.9 | 1.2 | 0.9 | 1.1 | 1.0 | 1.1 | 0.6 |
| Winc | 1.1 | 1.4 | 1.0 | 14.5 | 1.0 | 1.0 | 0.6 |
| Wbus | 1.6 | 1.4 | 1.1 | 14.6 | 1.2 | 1.1 | 0.8 |
| WAA | 2.0 | | | | | | |
| CarR | 2.3 | 2.0 | 1.3 | 15.1 | 4.0 | 3.2 | |
| MtrS | 1.0 | | | 9 | 1.1 | | |
| PubT | 1.6 | | | 14.6 | 1.1 | | |
| ReEd | | 1.5 | 0.9 | 8.9 | 1.2 | 1.2 | 0.8 |
| Tax | | 1.6 | 1.4 | 9.2 | 1.2 | | |
| IndT | | 1.7 | 1.2 | 9.0 | 1.2 | 0.9 | 0.6 |
| ChiT | | 1.4 | 1.1 | 9.1 | 1.1 | 1.0 | 0.6 |
| IndI | | 1.7 | 1.3 | 9.2 | 1.3 | 1.2 | 0.7 |
| ChiI | | 1.6 | 1.1 | 9.2 | 1.4 | 1.4 | 1.1 |
| GovH | | 1 | | 9.1 | 1.1 | | |
| NatS | | 0.9 | | | 0.2 | | |
| PosC | | 1.3 | | | 1.5 | | |
| RwID | | | 4.7 | 8.0 | 9.5 | 6.9 | 6.6 |
| Aany | | | 0.9 | 8.7 | 0.9 | 1.3 | 1.0 |
| Asp | | | 1.1 | 9.2 | 1.2 | 1.1 | 0.8 |
| pNZ1 | | | | 17.3 | 5.8 | 3.4 | 2.1 |
| IFF | | | | 32.4 | | | |
| pMan | | | | | 5.9 | 3.6 | |
| pCon | | | | | 6.1 | 3.6 | |
| SSM | | | | | 1.2 | 1.2 | 0.7 |

| | | | | | | | |
|---|---|---|---|---|---|---|---|
| Euth | | | | | | 2.6 | |
| 3stk | | | | | | 1.2 | |
| SexO | | | | | | 1.1 | |
| PayJ | | | | | | 1.2 | |
| PayP | | | | | | 1.2 | |
| IncR | | | | | | 1.4 | 0.7 |
| ForF | | | | | | 1.2 | 0.9 |
| ForR | | | | | | 1.5 | 0.8 |
| GMO | | | | | | 1.1 | 0.6 |
| Minimum | 0.9 | 0.9 | 0.9 | 1.1 | 0.2 | 0.8 | 0.4 |
| Maximum | 7.8 | 6.7 | 5.9 | 32.4 | 9.5 | 7.2 | 6.6 |
| Mean | 3.2 | 2.5 | 2.0 | 10.0 | 2.6 | 2.0 | 1.4 |

**Table S2. Percentage of sample that can be dropped and still retain a correlation of at least .75 between the original and re-estimated centrality estimates.**

| | Stability | | |
|---|---|---|---|
| Wave | Betweenness | Closeness | Strength |
| 1 | 75.0% | 75.0% | 75.0% |
| 2 | 75.0% | 75.0% | 75.0% |
| 3 | 75.0% | 75.0% | 75.0% |
| 4 | 75.0% | 75.0% | 75.0% |
| 5 | 75.0% | 75.0% | 75.0% |
| 6 | 75.0% | 75.0% | 75.0% |
| 7 | 75.0% | 75.0% | 75.0% |

**Table S3. Full results of the ANOVAs reported in the text that test if symbolic or operational components of the belief system are more central.**

| Strength | *df* | SS | MS | *F* | *p* |
|---|---|---|---|---|---|
| Item Type | 1 | 1.672 | 1.672 | 35.010 | 0.000 |
| Wave | 6 | 3.607 | 0.601 | 12.587 | 0.000 |
| Wave*Item Type | 6 | 0.093 | 0.016 | 0.326 | 0.923 |
| Residual | 204 | 9.742 | 0.048 | | |
| Closeness | *df* | SS | MS | *F* | *p* |
| Item Type | 1 | 0.073 | 0.073 | 172.536 | <0.001 |
| Wave | 6 | 0.528 | 0.088 | 209.355 | <0.001 |
| Wave*Item Type | 6 | 0.005 | 0.001 | 2.014 | 0.065 |
| Residual | 204 | 0.086 | 0.000 | | |
| Betweenness | *df* | SS | MS | *F* | *p* |
| Item Type | 1 | 17558.980 | 17558.980 | 40.191 | <0.001 |
| Wave | 6 | 7290.355 | 1215.059 | 2.781 | 0.013 |
| Wave*Item Type | 6 | 892.901 | 148.817 | 0.341 | 0.915 |
| Residual | 204 | 89126.060 | 436.892 | | |

**Table S4. Full results of the ANOVAs reported in the text that test if symbolic or operational components of the belief system are closer to behaviors.**

| Environment Routine | *df* | SS | MS | *F* | *p* |
|---|---|---|---|---|---|
| Item Type | 1 | 798.945 | 798.945 | 27.452 | <0.001 |
| Wave | 1 | 8.464 | 8.464 | 0.291 | 0.592 |
| Wave*Item Type | 1 | 0.030 | 0.030 | 0.001 | 0.974 |
| Residual | 55 | 1600.704 | 29.104 | | |
| Environment Sacrifice | *df* | SS | MS | *F* | *p* |
| Item Type | 1 | 875.417 | 875.417 | 30.319 | <0.001 |
| Wave | 1 | 10.338 | 10.338 | 0.358 | 0.552 |
| Wave*Item Type | 1 | 0.200 | 0.200 | 0.007 | 0.934 |
| Residual | 55 | 1588.049 | 28.874 | | |
| Religious Behavior | *df* | SS | MS | *F* | *p* |
| Item Type | 1 | 312.590 | 312.590 | 5.921 | 0.018 |
| Wave | 1 | 112.383 | 112.383 | 2.129 | 0.150 |
| Wave*Item Type | 1 | 20.176 | 20.176 | 0.382 | 0.539 |
| Residual | 55 | 2903.758 | 52.796 | | |
| Voting Behavior | *df* | SS | MS | *F* | *p* |
| Item Type | 1 | 1115.265 | 1115.265 | 61.208 | <0.001 |
| Wave | 1 | 3.222 | 3.222 | 0.177 | 0.676 |
| Wave*Item Type | 1 | 1.652 | 1.652 | 0.091 | 0.764 |
| Residual | 55 | 1002.150 | 18.221 | | |

**Table S5. Results of the ANOVAs testing if symbolic or operational components of the belief system are more central using standardized measures of centrality.**

| Strength | *df* | SS | MS | *F* | *p* |
|---|---|---|---|---|---|
| Item Type | 1 | 2.576 | 2.576 | 31.509 | <0.001 |
| Wave | 6 | 0.005 | 0.001 | 0.011 | 1.000 |
| Wave*Item Type | 6 | 0.117 | 0.019 | 0.238 | 0.964 |
| Residual | 204 | 16.676 | 0.082 | | |
| Closeness | *df* | SS | MS | *F* | *p* |
| Item Type | 1 | 8.563 | 8.563 | 163.636 | <0.001 |
| Wave | 6 | 0.018 | 0.003 | 0.057 | 0.999 |
| Wave*Item Type | 6 | 0.116 | 0.019 | 0.371 | 0.897 |
| Residual | 204 | 10.676 | 0.052 | | |
| Betweenness | *df* | SS | MS | *F* | *p* |
| Item Type | 1 | 2.385 | 2.385 | 31.401 | <0.001 |
| Wave | 6 | 0.007 | 0.001 | 0.016 | 1.000 |
| Wave*Item Type | 6 | 0.211 | 0.035 | 0.463 | 0.835 |
| Residual | 204 | 15.492 | 0.076 | | |

**Table S6. Results of the ANOVAs testing if symbolic or operational components of the belief system are more central using items available in at least six waves.**

| Strength | *df* | SS | MS | *F* | *p* |
|---|---|---|---|---|---|
| Item Type | 1 | 0.148 | 0.148 | 3.353 | 0.069 |
| Wave | 6 | 0.709 | 0.118 | 2.686 | 0.017 |
| Wave*Item Type | 6 | 0.025 | 0.004 | 0.094 | 0.997 |
| Residual | 139 | 6.117 | 0.044 | | |
| **Closeness** | *df* | SS | MS | *F* | *p* |
| Item Type | 1 | 0.164 | 0.164 | 122.525 | <0.001 |
| Wave | 6 | 0.283 | 0.047 | 35.221 | 0.000 |
| Wave*Item Type | 6 | 0.004 | 0.001 | 0.485 | 0.819 |
| Residual | 139 | 0.186 | 0.001 | | |
| **Betweenness** | *df* | SS | MS | *F* | *p* |
| Item Type | 1 | 4440.012 | 4440.012 | 26.794 | <0.001 |
| Wave | 6 | 672.157 | 112.026 | 0.676 | 0.669 |
| Wave*Item Type | 6 | 379.513 | 63.252 | 0.382 | 0.890 |
| Residual | 139 | 23033.980 | 165.712 | | |

**Table S7. Results of the ANOVAs testing if symbolic or operational components of the belief system are closer to behaviors using items available in at least six waves.**

| Environment Routine | *df* | SS | MS | *F* | *p* |
|---|---|---|---|---|---|
| Item Type | 1 | 660.049 | 660.049 | 31.418 | <0.001 |
| Wave | 1 | 9.791 | 9.791 | 0.466 | 0.499 |
| Wave*Item Type | 1 | 0.121 | 0.121 | 0.006 | 0.940 |
| Residual | 41 | 861.349 | 21.009 | | |
| **Environment Sacrifice** | *df* | SS | MS | *F* | *p* |
| Item Type | 1 | 686.639 | 686.639 | 33.176 | <0.001 |
| Wave | 1 | 17.573 | 17.573 | 0.849 | 0.362 |
| Wave*Item Type | 1 | 0.020 | 0.020 | 0.001 | 0.976 |
| Residual | 41 | 848.569 | 20.697 | | |
| **Religious Behavior** | *df* | SS | MS | *F* | *p* |
| Item Type | 1 | 269.412 | 269.412 | 8.892 | 0.005 |
| Wave | 1 | 13.636 | 13.636 | 0.450 | 0.506 |
| Wave*Item Type | 1 | 0.024 | 0.024 | 0.001 | 0.978 |
| Residual | 41 | 1242.231 | 30.298 | | |
| **Voting Behavior** | *df* | SS | MS | *F* | *p* |
| Item Type | 1 | 622.120 | 622.120 | 40.563 | <0.001 |
| Wave | 1 | 12.362 | 12.362 | 0.806 | 0.375 |
| Wave*Item Type | 1 | 4.689 | 4.689 | 0.306 | 0.583 |
| Residual | 41 | 628.820 | 15.337 | | |

**Table S8. Results of permutation tests if high/low political knowledge and high/low education networks differ in overall levels of strength.**

| | Political Knowledge Differences | | Education Differences | |
|---|---|---|---|---|
| | S | p-value | S | p-value |
| 2009 Wave | 0.767 | 0.103 | 0.333 | 0.729 |
| 2010 Wave | 0.437 | 0.469 | 0.327 | 0.791 |
| 2011 Wave | 0.177 | 0.631 | 0.438 | 0.236 |
| 2012 Wave | 0.775 | 0.218 | 0.384 | 0.805 |
| 2013 Wave | 0.098 | 0.889 | 0.194 | 0.910 |
| 2014 Wave | 1.956 | 0.008 | 1.402 | 0.114 |
| 2015 Wave | 0.805 | 0.175 | 0.439 | 0.594 |

**Table S9. Results of the ANOVA testing if symbolic or operational components of the belief system differ in strength for people high and low in political knowledge.**

| Strength | *df* | SS | MS | *F* | *p* |
|---|---|---|---|---|---|
| Item Type | 1 | 2.036 | 2.036 | 36.942 | <0.001 |
| Wave | 6 | 1.342 | 0.224 | 4.059 | 0.001 |
| Political Knowledge | 1 | 0.213 | 0.213 | 3.865 | 0.050 |
| Item Type*Wave | 6 | 0.137 | 0.023 | 0.415 | 0.869 |
| Item Type*Political Knowledge | 1 | 0.009 | 0.009 | 0.169 | 0.681 |
| Wave*Political Knowledge | 6 | 0.124 | 0.021 | 0.376 | 0.894 |
| Item Type*Wave*Political Knowledge | 6 | 0.042 | 0.007 | 0.126 | 0.993 |
| Residuals | 408 | 22.485 | 0.055 | | |

**Table S10. Results of the ANOVA testing if symbolic or operational components of the belief system differ in closeness for people high and low in political knowledge.**

| Closeness | *df* | SS | MS | *F* | *p* |
|---|---|---|---|---|---|
| Item Type | 1 | 0.111 | 0.111 | 238.238 | <0.001 |
| Wave | 6 | 1.026 | 0.171 | 368.721 | <0.001 |
| Political Knowledge | 1 | 0.0003 | 0.0003 | 0.683 | 0.409 |
| Item Type*Wave | 6 | 0.008 | 0.001 | 3.003 | 0.007 |
| Item Type*Political Knowledge | 1 | 0.001 | 0.001 | 2.359 | 0.125 |
| Wave*Political Knowledge | 6 | 0.016 | 0.003 | 5.834 | <0.001 |
| Item Type*Wave*Political Knowledge | 6 | 0.001 | 0.0002 | 0.341 | 0.915 |
| Residuals | 408 | 0.189 | 0.0005 | | |

**Table S11. Results of the ANOVA testing if symbolic or operational components of the belief system differ in betweenness for people high and low in political knowledge.**

| Betweenness | *df* | SS | MS | *F* | *p* |
|---|---|---|---|---|---|
| Item Type | 1 | 42591.040 | 42591.040 | 73.781 | <0.001 |
| Wave | 6 | 18135.950 | 3022.659 | 5.236 | <0.001 |
| Political Knowledge | 1 | 519.670 | 519.670 | 0.900 | 0.343 |
| Item Type*Wave | 6 | 7160.018 | 1193.336 | 2.067 | 0.056 |
| Item Type*Political Knowledge | 1 | 3220.485 | 3220.485 | 5.579 | 0.019 |
| Wave*Political Knowledge | 6 | 292.771 | 48.795 | 0.085 | 0.998 |
| Item Type*Wave*Political Knowledge | 6 | 822.462 | 137.077 | 0.237 | 0.964 |
| Residuals | 408 | 235523.800 | 577.264 | | |

**Table S12. Results of the ANOVA testing if symbolic or operational components of the belief system differ in strength for people high and low in political knowledge as determined by quartiles.**

| Strength | *df* | SS | MS | *F* | *p* |
|---|---|---|---|---|---|
| Item Type | 1 | 1.584 | 1.584 | 25.940 | <0.001 |
| Wave | 6 | 1.884 | 0.314 | 5.141 | <0.001 |
| Political Knowledge | 1 | 0.384 | 0.384 | 6.282 | 0.013 |
| Item Type*Wave | 6 | 0.188 | 0.031 | 0.512 | 0.799 |
| Item Type*Political Knowledge | 1 | 0.032 | 0.032 | 0.526 | 0.469 |
| Wave*Political Knowledge | 6 | 0.213 | 0.035 | 0.581 | 0.746 |
| Item Type*Wave*Political Knowledge | 6 | 0.131 | 0.022 | 0.357 | 0.905 |
| Residuals | 408 | 24.916 | 0.061 | | |

**Table S13. Results of the ANOVA testing if symbolic or operational components of the belief system differ in closeness for people high and low in political knowledge as determined by quartiles.**

| Closeness | *df* | SS | MS | *F* | *p* |
|---|---|---|---|---|---|
| Item Type | 1 | 0.100 | 0.100 | 220.853 | <0.001 |
| Wave | 6 | 0.932 | 0.155 | 341.413 | <0.001 |
| Political Knowledge | 1 | 0.001 | 0.001 | 3.167 | 0.076 |
| Item Type*Wave | 6 | 0.009 | 0.001 | 3.157 | 0.005 |
| Item Type*Political Knowledge | 1 | 0.0005 | 0.0005 | 1.033 | 0.310 |
| Wave*Political Knowledge | 6 | 0.014 | 0.002 | 5.285 | 0.000 |
| Item Type*Wave*Political Knowledge | 6 | 0.002 | 0.0003 | 0.740 | 0.618 |
| Residuals | 408 | 0.186 | 0.0005 | | |

**Table S14. Results of the ANOVA testing if symbolic or operational components of the belief system differ in betweenness for people high and low in political knowledge as determined by quartiles.**

| Betweenness | *df* | SS | MS | *F* | *p* |
|---|---|---|---|---|---|
| Item Type | 1 | 46662.860 | 46662.860 | 77.158 | 0.000 |
| Wave | 6 | 17001.420 | 2833.571 | 4.685 | 0.000 |
| Political Knowledge | 1 | 747.801 | 747.801 | 1.237 | 0.267 |
| Item Type*Wave | 6 | 3760.836 | 626.806 | 1.036 | 0.401 |
| Item Type*Political Knowledge | 1 | 1399.748 | 1399.748 | 2.315 | 0.129 |
| Wave*Political Knowledge | 6 | 467.950 | 77.992 | 0.129 | 0.993 |
| Item Type*Wave*Political Knowledge | 6 | 1176.468 | 196.078 | 0.324 | 0.924 |
| Residuals | 408 | 246745.900 | 604.769 | | |

**Table S15. Results of the ANOVA testing if symbolic or operational components of the belief system are closer to behavior for people high and low in political knowledge.**

| Environment Routine | *df* | SS | MS | *F* | *p* |
|---|---|---|---|---|---|
| Item Type | 1 | 1448.840 | 1448.840 | 42.937 | <0.001 |
| Wave | 1 | 44.942 | 44.942 | 1.332 | 0.251 |
| Political Knowledge | 1 | 141.755 | 141.755 | 4.201 | 0.043 |
| Item Type*Wave | 1 | 0.385 | 0.385 | 0.011 | 0.915 |
| Item Type*Political Knowledge | 1 | 21.824 | 21.824 | 0.647 | 0.423 |
| Wave*Political Knowledge | 1 | 147.164 | 147.164 | 4.361 | 0.039 |
| Item Type*Wave*Political Knowledge | 1 | 2.305 | 2.305 | 0.068 | 0.794 |
| Residual | 110 | 3711.792 | 33.744 | | |
| **Environment Sacrifice** | *df* | SS | MS | *F* | *p* |
| Item Type | 1 | 1451.547 | 1451.547 | 43.750 | <0.001 |
| Wave | 1 | 102.595 | 102.595 | 3.092 | 0.081 |
| Political Knowledge | 1 | 511.350 | 511.350 | 15.412 | <0.001 |
| Item Type*Wave | 1 | 0.230 | 0.230 | 0.007 | 0.934 |
| Item Type*Political Knowledge | 1 | 27.554 | 27.554 | 0.830 | 0.364 |
| Wave*Political Knowledge | 1 | 208.600 | 208.600 | 6.287 | 0.014 |
| Item Type*Wave*Political Knowledge | 1 | 1.218 | 1.218 | 0.037 | 0.848 |
| Residual | 110 | 3649.606 | 33.178 | | |
| **Religious Behavior** | *df* | SS | MS | *F* | *p* |
| Item Type | 1 | 789.434 | 789.434 | 16.582 | <0.001 |
| Wave | 1 | 24.962 | 24.962 | 0.524 | 0.471 |
| Political Knowledge | 1 | 269.528 | 269.528 | 5.661 | 0.019 |
| Item Type*Wave | 1 | 143.714 | 143.714 | 3.019 | 0.085 |
| Item Type*Political Knowledge | 1 | 27.382 | 27.382 | 0.575 | 0.450 |
| Wave*Political Knowledge | 1 | 190.121 | 190.121 | 3.994 | 0.048 |
| Item Type*Wave*Political Knowledge | 1 | 0.001 | 0.001 | 0.000 | 0.997 |
| Residual | 110 | 5236.834 | 47.608 | | |
| **Voting Behavior** | *df* | SS | MS | *F* | *p* |
| Item Type | 1 | 2748.065 | 2748.065 | 131.206 | <0.001 |
| Wave | 1 | 13.403 | 13.403 | 0.640 | 0.425 |
| Political Knowledge | 1 | 1.200 | 1.200 | 0.057 | 0.811 |
| Item Type*Wave | 1 | 5.541 | 5.541 | 0.265 | 0.608 |
| Item Type*Political Knowledge | 1 | 1.515 | 1.515 | 0.072 | 0.788 |
| Wave*Political Knowledge | 1 | 17.607 | 17.607 | 0.841 | 0.361 |
| Item Type*Wave*Political Knowledge | 1 | 3.898 | 3.898 | 0.186 | 0.667 |
| Residual | 110 | 2303.905 | 20.945 | | |

**Table S16. Results of the ANOVA testing if symbolic or operational components of the belief system are closer to behavior for people high and low in education.**

| Environment Routine | *df* | SS | MS | *F* | *p* |
|---|---|---|---|---|---|
| Item Type | 1 | 1011.182 | 1011.182 | 28.458 | <0.001 |
| Wave | 1 | 29.251 | 29.251 | 0.823 | 0.366 |
| Education | 1 | 145.850 | 145.850 | 4.105 | 0.045 |
| Item Type*Wave | 1 | 2.143 | 2.143 | 0.060 | 0.806 |
| Item Type*Education | 1 | 0.913 | 0.913 | 0.026 | 0.873 |
| Wave*Education | 1 | 154.567 | 154.567 | 4.350 | 0.039 |
| Item Type*Wave*Education | 1 | 4.689 | 4.689 | 0.132 | 0.717 |
| Residual | 110 | 3908.596 | 35.533 | | |
| Environment Sacrifice | *df* | SS | MS | *F* | *p* |
| Item Type | 1 | 1213.769 | 1213.769 | 36.255 | <0.001 |
| Wave | 1 | 19.295 | 19.295 | 0.576 | 0.449 |
| Education | 1 | 154.412 | 154.412 | 4.612 | 0.034 |
| Item Type*Wave | 1 | 0.614 | 0.614 | 0.018 | 0.893 |
| Item Type*Education | 1 | 1.356 | 1.356 | 0.041 | 0.841 |
| Wave*Education | 1 | 198.258 | 198.258 | 5.922 | 0.017 |
| Item Type*Wave*Education | 1 | 1.739 | 1.739 | 0.052 | 0.820 |
| Residual | 110 | 3682.672 | 33.479 | | |
| Religious Behavior | *df* | SS | MS | *F* | *p* |
| Item Type | 1 | 473.325 | 473.325 | 8.539 | 0.004 |
| Wave | 1 | 345.644 | 345.644 | 6.236 | 0.014 |
| Education | 1 | 202.701 | 202.701 | 3.657 | 0.058 |
| Item Type*Wave | 1 | 2.592 | 2.592 | 0.047 | 0.829 |
| Item Type*Education | 1 | 31.228 | 31.228 | 0.563 | 0.454 |
| Wave*Education | 1 | 254.012 | 254.012 | 4.583 | 0.035 |
| Item Type*Wave*Education | 1 | 5.501 | 5.501 | 0.099 | 0.753 |
| Residual | 110 | 6097.145 | 55.429 | | |
| Voting Behavior | *df* | SS | MS | *F* | *p* |
| Item Type | 1 | 2050.745 | 2050.745 | 99.525 | <0.001 |
| Wave | 1 | 63.557 | 63.557 | 3.084 | 0.082 |
| Education | 1 | 87.819 | 87.819 | 4.262 | 0.041 |
| Item Type*Wave | 1 | 13.328 | 13.328 | 0.647 | 0.423 |
| Item Type*Education | 1 | 26.393 | 26.393 | 1.281 | 0.260 |
| Wave*Education | 1 | 24.696 | 24.696 | 1.199 | 0.276 |
| Item Type*Wave*Education | 1 | 10.840 | 10.840 | 0.526 | 0.470 |
| Residual | 110 | 2266.587 | 20.605 | | |

**Table S17. Results of the ANOVA testing if symbolic or operational components of the belief system differ in strength for people high and low in education.**

| Strength | *df* | SS | MS | *F* | *p* |
|---|---|---|---|---|---|
| Item Type | 1 | 2.847 | 2.847 | 59.794 | <0.001 |
| Wave | 6 | 5.807 | 0.968 | 20.327 | <0.001 |
| Education | 1 | 0.026 | 0.026 | 0.553 | 0.457 |
| Item Type*Wave | 6 | 0.175 | 0.029 | 0.613 | 0.720 |
| Item Type*Education | 1 | 0.003 | 0.003 | 0.068 | 0.795 |
| Wave*Education | 6 | 0.127 | 0.021 | 0.443 | 0.850 |
| Item Type*Wave*Education | 6 | 0.031 | 0.005 | 0.108 | 0.996 |
| Residuals | 408 | 19.427 | 0.048 | | |

**Table S18. Results of the ANOVA testing if symbolic or operational components of the belief system differ in closeness for people high and low in education.**

| Closeness | *df* | SS | MS | *F* | *p* |
|---|---|---|---|---|---|
| Item Type | 1 | 0.145 | 0.145 | 356.218 | <0.001 |
| Wave | 6 | 0.938 | 0.156 | 384.601 | <0.001 |
| Education | 1 | 0.00005 | 0.00005 | 0.115 | 0.735 |
| Item Type*Wave | 6 | 0.009 | 0.001 | 3.595 | 0.002 |
| Item Type*Education | 1 | 0.0002 | 0.0002 | 0.508 | 0.477 |
| Wave*Education | 6 | 0.003 | 0.0004 | 1.025 | 0.408 |
| Item Type*Wave*Education | 6 | 0.0004 | 0.0001 | 0.149 | 0.989 |
| Residuals | 408 | 0.166 | 0.0004 | | |

**Table S19. Results of the ANOVA testing if symbolic or operational components of the belief system differ in betweenness for people high and low in education.**

| Betweenness | *df* | SS | MS | *F* | *p* |
|---|---|---|---|---|---|
| Item Type | 1 | 41943.110 | 41943.110 | 86.438 | <0.001 |
| Wave | 6 | 13312.760 | 2218.794 | 4.573 | <0.001 |
| Education | 1 | 93.587 | 93.587 | 0.193 | 0.661 |
| Item Type*Wave | 6 | 1239.115 | 206.519 | 0.426 | 0.862 |
| Item Type*Education | 1 | 555.962 | 555.962 | 1.146 | 0.285 |
| Wave*Education | 6 | 261.333 | 43.556 | 0.090 | 0.997 |
| Item Type*Wave*Education | 6 | 481.980 | 80.330 | 0.166 | 0.986 |
| Residuals | 408 | 197977.400 | 485.239 | | |

**Table S20. Results of the ANOVAs testing if specific subtypes of symbolic and operational components of the belief system are more central.**

| Strength | *df* | SS | MS | *F* | *p* |
|---|---|---|---|---|---|
| Item Subtype | 3 | 2.012 | 0.671 | 14.756 | <0.001 |
| Wave | 6 | 3.351 | 0.558 | 12.288 | <0.001 |
| Wave*Item Subtype | 18 | 0.331 | 0.018 | 0.405 | 0.986 |
| Residual | 179 | 8.136 | 0.045 | | |
| **Closeness** | *df* | SS | MS | *F* | *p* |
| Item Subtype | 3 | 0.074 | 0.025 | 68.140 | <0.001 |
| Wave | 6 | 0.504 | 0.084 | 231.281 | <0.001 |
| Wave*Item Subtype | 18 | 0.010 | 0.001 | 1.478 | 0.102 |
| Residual | 179 | 0.065 | 0.0004 | | |
| **Betweenness** | *df* | SS | MS | *F* | *p* |
| Item Subtype | 3 | 18085.180 | 6028.394 | 13.438 | <0.001 |
| Wave | 6 | 7255.586 | 1209.264 | 2.696 | 0.016 |
| Wave*Item Subtype | 18 | 3667.125 | 203.729 | 0.454 | 0.973 |
| Residual | 179 | 80298.990 | 448.598 | | |

**Table S21. P-values from pair-wise comparisons between specific types of symbolic and operational components. This is a follow-up to the main effect of Item Type in Table S14.**

| Subtype Comparison | Strength *p*-value | Closeness *p*-value | Betweenness *p*-value |
|---|---|---|---|
| Ideo ID-Party ID | 0.026 | 0.257 | 0.272 |
| Social Policy-Party ID | <0.001 | <0.001 | <0.001 |
| Economic Policy-Party ID | <0.001 | <0.001 | <0.001 |
| Social Policy-Ideo ID | 0.898 | 0.062 | 0.017 |
| Economic Policy-Ideo ID | 0.225 | 0.327 | 0.041 |
| Economic Policy-Social Policy | 0.040 | 0.088 | 0.505 |

**Supplemental References**

Converse, P. E. (1964). The nature of belief systems in mass publics. In D. E. Apter (Ed.), *Ideology and discontent* (pp. 206-261). New York, NY: The Free Press.

Electoral Commission. (2018). *MMP Voting System*. Wellington, New Zealand. Retrieved from http://www.elections.org.nz/

Epskamp, S., Borsboom, D., & Fried, E. I. (2017). Estimating psychological networks and their accuracy: A tutorial paper. *Behavior Research Methods*, 1-18.

Erdös, P., & Rényi, A. (1959). On random graphs, I. *Publicationes Mathematicae (Debrecen), 6*, 290-297.

Federico, C. M., & Schneider, M. C. (2007). Political expertise and the use of ideology: Moderating effects of evaluative motivation. *Public Opinion Quarterly, 71*, 221-252.

Greaves, L., Osborne, D., Sengupta, N. K., Milojev, P., & Sibley, C. G. (2014). Politics and post-colonial ideology: Historical negation and symbolic exclusion predict political party preference. *New Zealand Journal of Psychology, 43*(3), 39-54.

Jones, P. J., Mair, P., & McNally, R. J. (2018). Visualizing Psychological Networks: A Tutorial in R. *Frontiers in Psychology*, *9*.

Newton, H. J., Sibley, C. G., & Osborne, D. (2018). The predictive power of post-colonial ideologies: Historical negation and symbolic exclusion undermine support for resource-based bicultural policies. *International Journal of Intercultural Relations, 62*, 23-33. doi:10.1016/j.ijintrel.2017.03.005

Orange, C. (2011). *The Treaty of Waitangi* (2nd ed.). Wellington, New Zealand: Bridget Williams Books.

Osborne, D., Yogeeswaran, K., & Sibley, C. G. (2017). Culture-specific ideologies undermine collective action support: Examining the legitimizing effects of postcolonial belief systems. *Group Processes & Intergroup Relations, 20*(3), 333-349. doi:10.1177/1368430216682352

Revelle, W. (2017) psych: Procedures for Personality and Psychological Research, Northwestern University, Evanston, Illinois, USA, https://CRAN.R-project.org/package=psych Version = 1.7.8.

Sibley, C. G., & Osborne, D. (2016). Ideology and post-colonial society. *Advances in Political Psychology, 37*(1), 115-161. doi:10.1111/pops.12323

Statistics New Zealand. (2018). *Population*. Wellington, New Zealand. Retrieved from http://www.stats.govt.nz/

van Borkulo, C. D., Epskamp, S., & Millner, A. (2016). NetworkComparisonTest: Statistical comparison of two networks based on three invariance measures. R package version 2.0.1. https://CRAN.R-project.org/package=NetworkComparisonTest

Yin, J., & Li, H. (2011). A sparse conditional gaussian graphical model for analysis of genetical genomics data. *The Annals of Applied Statistics, 5*, 2630.

Zaller, J. (1992). *The nature and origins of mass opinion*. Cambridge University Press.
