# Supplementary material for: What Is Central to Political Belief System Networks?
Source: Pers Soc Psychol Bull. 2019 Jan 28;45(9):1352–64. doi: 10.1177/0146167218824354 (PMC6676336; doi:10.1177/0146167218824354)
Supplement: Brandt_Online_Appendix – Supplemental material for What Is Central to Political Belief System Networks? [file Brandt_Online_Appendix.pdf]

Methods Reporting

The data from this study come from the New Zealand Attitudes and Values Survey. Detailed methodological information – including questionnaires, sampling frame, and quality checks – can be found here: https://www.psych.auckland.ac.nz/en/about/our-research/research-groups/new-zealand-attitudes-and-values-study/nzavs-tech-docs.html

The questionnaires for the data used in this study are appended.
