# Supplementary material for: What Is Central to Political Belief System Networks?
Source: Pers Soc Psychol Bull. 2019 Jan 28;45(9):1352–64. doi: 10.1177/0146167218824354 (PMC6676336; doi:10.1177/0146167218824354)
Supplement: PSPB824354_Supplemental_Material_CLN – Supplemental material for What Is Central to Political Belief System Networks? [file PSPB824354_Supplemental_Material_CLN.docx]

**Appendix**

**List of additional information available in supplemental materials**

- Summary of Political Situation in New Zealand
- Network Stability
- Centrality Stability
- Simulated Specificity, Sensitivity, and Stability
- Complete Results from the Main Text
- Standardizing Centrality Estimates
- Robustness Checks on the Inclusion of Nodes
- Political Knowledge & Education Analyses
- Different Subtypes of Symbolic and Operational Components
- Supplemental References

**List of Supplemental Figures**

- Figure S1. Belief system networks with symbolic (gold nodes) and operational (blue nodes) components for Wave 1 – Wave 4.
- Figure S2. Belief system networks with symbolic (gold nodes) and operational (blue nodes) components for Wave 5 – Wave 7.
- Figure S3. Edge estimates (red dots) with bootstrapped 95% confidence interval (gray shaded region) for Wave 1.
- Figure S4. Edge estimates (red dots) with bootstrapped 95% confidence interval (gray shaded region) for Wave 2.
- Figure S5. Edge estimates (red dots) with bootstrapped 95% confidence interval (gray shaded region) for Wave 3.
- Figure S6. Edge estimates (red dots) with bootstrapped 95% confidence interval (gray shaded region) for Wave 4.
- Figure S7. Edge estimates (red dots) with bootstrapped 95% confidence interval (gray shaded region) for Wave 5.
- Figure S8. Edge estimates (red dots) with bootstrapped 95% confidence interval (gray shaded region) for Wave 6.
- Figure S9. Edge estimates (red dots) with bootstrapped 95% confidence interval (gray shaded region) for Wave 7.
- Figure S10. Network specificity, sensitivity, and edge correlations for simulated networks.
- Figure S11. Correlations between simulated networks and true networks centrality estimates.
- Figure S12. Lower connectivity “true” networks used for the simulation study.
- Figure S13. Higher connectivity “true” networks used for the simulation study. Solid blue edges are positive.
- Figure S14. Edge weight bias and correlations comparing the edges in the true networks to the edges in the simulated networks for each of the three estimation methods.
- Figure S15. Correlations comparing centrality estimates in the true networks to the edges in the simulated networks for each of the three estimation methods.
- Figure S16. Symbolic components are higher in strength, closeness, and betweenness centrality than operational components across the belief system networks using standardized measures of centrality.
- Figure S17. Symbolic components are higher in closeness and betweenness centrality than operational components across the belief system networks that only include nodes found in at least 6 of the waves.
- Figure S18. Symbolic components are higher in closeness and betweenness centrality than operational components across the belief system networks that only include nodes found in at least 6 of the waves and using standardized measures of centrality.
- Figure S19. Symbolic components are more closely connected (have shorter paths) with behaviors than operational components in networks that only include nodes found in at least 6 of the waves.
- Figure S20. Belief system networks with symbolic (gold nodes) and operational (blue nodes) components for Waves 1 & 2 for people with high and low levels of political knowledge.
- Figure S21. Belief system networks with symbolic (gold nodes) and operational (blue nodes) components for Waves 3 & 4 for people with high and low levels of political knowledge.
- Figure S22. Belief system networks with symbolic (gold nodes) and operational (blue nodes) components for Waves 5 & 6 for people with high and low levels of political knowledge.
- Figure S23. Belief system networks with symbolic (gold nodes) and operational (blue nodes) components for Wave 7 for people with high and low levels of political knowledge.
- Figure S24. Symbolic components are higher in centrality than operational components across the belief system networks.
- Figure S25. Symbolic components are higher in centrality than operational components across the belief system networks.
- Figure S26. Symbolic components are more closely connected (have shorter paths) with behaviors than operational components for people with both high and low levels of political knowledge.
- Figure S27. Symbolic components are more closely connected (have shorter paths) with behaviors than operational components for people with both high and low levels of education.
- Figure S28. Belief system networks with symbolic (gold nodes) and operational (blue nodes) components for Waves 1 & 2 for people with high and low levels of education.
- Figure S29. Belief system networks with symbolic (gold nodes) and operational (blue nodes) components for Waves 3 & 4 for people with high and low levels of education.
- Figure S30. Belief system networks with symbolic (gold nodes) and operational (blue nodes) components for Waves 5 & 6 for people with high and low levels of education.
- Figure S31. Belief system networks with symbolic (gold nodes) and operational (blue nodes) components for Wave 7 for people with high and low levels of education.
- Figure S32. Symbolic components are higher in centrality than operational components across the belief system networks
- Figure S33. Symbolic components are higher in centrality than operational components across the belief system networks.

**List of Supplemental Tables**

- Table S1. Percentage of missing data per item per wave.
- Table S2. Percentage of sample that can be dropped and still retain a correlation of at least .75 between the original and re-estimated centrality estimates.
- Table S3. Full results of the ANOVAs reported in the text that test if symbolic or operational components of the belief system are more central.
- Table S4. Full results of the ANOVAs reported in the text that test if symbolic or operational components of the belief system are closer to behaviors.
- Table S5. Results of the ANOVAs testing if symbolic or operational components of the belief system are more central using standardized measures of centrality.
- Table S6. Results of the ANOVAs testing if symbolic or operational components of the belief system are more central using items available in at least six waves.
- Table S7. Results of the ANOVAs testing if symbolic or operational components of the belief system are closer to behaviors using items available in at least six waves.
- Table S8. Results of permutation tests if high/low political knowledge and high/low education networks differ in overall levels of strength.
- Table S9. Results of the ANOVA testing if symbolic or operational components of the belief system differ in strength for people high and low in political knowledge.
- Table S10. Results of the ANOVA testing if symbolic or operational components of the belief system differ in closeness for people high and low in political knowledge.
- Table S11. Results of the ANOVA testing if symbolic or operational components of the belief system differ in betweenness for people high and low in political knowledge.
- Table S12. Results of the ANOVA testing if symbolic or operational components of the belief system differ in strength for people high and low in political knowledge as determined by quartiles.
- Table S13. Results of the ANOVA testing if symbolic or operational components of the belief system differ in closeness for people high and low in political knowledge as determined by quartiles.
- Table S14. Results of the ANOVA testing if symbolic or operational components of the belief system differ in betweenness for people high and low in political knowledge as determined by quartiles.
- Table S15. Results of the ANOVA testing if symbolic or operational components of the belief system are closer to behavior for people high and low in political knowledge.
- Table S16. Results of the ANOVA testing if symbolic or operational components of the belief system are closer to behavior for people high and low in education.
- Table S17. Results of the ANOVA testing if symbolic or operational components of the belief system differ in strength for people high and low in education.
- Table S18. Results of the ANOVA testing if symbolic or operational components of the belief system differ in closeness for people high and low in education.
- Table S19. Results of the ANOVA testing if symbolic or operational components of the belief system differ in betweenness for people high and low in education.
- Table S20. Results of the ANOVAs testing if specific subtypes of symbolic and operational components of the belief system are more central.
- Table S21. P-values from pair-wise comparisons between specific types of symbolic and operational components. This is a follow-up to the main effect of Item Type in Table S14.
